# A novel Ancestral Beijing sublineage of *Mycobacterium tuberculosis* suggests the transition site to Modern Beijing sublineages

**DOI:** 10.1038/s41598-019-50078-3

**Published:** 2019-09-23

**Authors:** Pravech Ajawatanawong, Hideki Yanai, Nat Smittipat, Areeya Disratthakit, Norio Yamada, Reiko Miyahara, Supalert Nedsuwan, Worarat Imasanguan, Pacharee Kantipong, Boonchai Chaiyasirinroje, Jiraporn Wongyai, Supada Plitphonganphim, Pornpen Tantivitayakul, Jody Phelan, Julian Parkhill, Taane G. Clark, Martin L. Hibberd, Wuthiwat Ruangchai, Panawun Palittapongarnpim, Tada Juthayothin, Yuttapong Thawornwattana, Wasna Viratyosin, Sissades Tongsima, Surakameth Mahasirimongkol, Katsushi Tokunaga, Prasit Palittapongarnpim

**Affiliations:** 1 Department of Microbiology, Faculty of Science, Mahidol University, Rama 6 Road, Bangkok, Thailand; 2 Fukujuji Hospital, Japan Anti-Tuberculosis Association (JATA), Kiyose, Japan; 3 National Center for Genetic Engineering and Biotechnology, National Science and Technology Development Agency, Phahonyothin Road, Pathumthani, Thailand; 4 Department of Medical Sciences, Ministry of Public Health, Tiwanon Road, Nonthaburi, Thailand; 5 Research Institute of Tuberculosis, JATA, Kiyose, Japan; 6 Present Address: Genome Medical Science Project, National Center for Global Health and Medicine, Tokyo, Japan; 7 Chiangrai Prachanukroh Hospital, Ministry of Public Health, Chiangrai, Thailand; 8 TB-HIV Research Foundation, Chiangrai, Thailand; 9 Department of Biostatistics, Faculty of Public Health, Mahidol University, Bangkok, Thailand; 10 Department of Oral Microbiology, Faculty of Dentistry, Mahidol University, Bangkok, Thailand; 11 London School of Hygiene and Tropical Medicine, London, UK; 12 Welcome Trust Sanger Institute, Hinxton, Cambridge, UK

**Keywords:** Classification and taxonomy, Coevolution, Bacteriology, Risk factors

## Abstract

Global *Mycobacterium tuberculosis* population comprises 7 major lineages. The Beijing strains, particularly the ones classified as Modern groups, have been found worldwide, frequently associated with drug resistance, younger ages, outbreaks and appear to be expanding. Here, we report analysis of whole genome sequences of 1170 *M*. *tuberculosis* isolates together with their patient profiles. Our samples belonged to Lineage 1–4 (L1–L4) with those of L1 and L2 being equally dominant. Phylogenetic analysis revealed several new or rare sublineages. Differential associations between sublineages of *M*. *tuberculosis* and patient profiles, including ages, ethnicity, HIV (human immunodeficiency virus) infection and drug resistance were demonstrated. The Ancestral Beijing strains and some sublineages of L4 were associated with ethnic minorities while L1 was more common in Thais. L2.2.1.Ancestral 4 surprisingly had a mutation that is typical of the Modern Beijing sublineages and was common in Akha and Lahu tribes who have migrated from Southern China in the last century. This may indicate that the evolutionary transition from the Ancestral to Modern Beijing sublineages might be gradual and occur in Southern China, where the presence of multiple ethnic groups might have allowed for the circulations of various co-evolving sublineages which ultimately lead to the emergence of the Modern Beijing strains.

## Introduction

*Mycobacterium tuberculosis* causes a serious health problem and premature mortality worldwide. Whole genome sequences (WGS) of *M*. *tuberculosis* provide detail information about its genetic variations and insights to its phenotypes and how it interacts with human^1–5^.

*M*. *tuberculosis* complex is classified into 7 lineages^1,6^, with different geographical distributions. The bacteria in Thailand belonged predominantly to lineages 1 or 2^3^. *M*. *tuberculosis* lineage 2 (L2) is common in Eastern Asia and has spread worldwide. The lineage comprises two sublineages, L2.1 and L2.2^7^. The former is found mainly in Southern China, particularly in Guangxi province, and is usually referred to as the proto-Beijing strain^7^. L2.2, or the Beijing strain as defined by spoligotyping, is composed of several sublineages broadly categorized into the Ancestral and Modern Beijing strains^8^. The Ancestral Beijing strains forms a phylogenetic cluster that exhibit a cascading feature with the Modern Beijing strains forming a clade at the end of the cascade. The latter has a star-shaped phylogeny, caused several outbreaks around the world and is associated with drug resistance in some countries^5,9^. They are common and appear to be expanding in many countries^5^ and, therefore, have been extensively studied. Their frequently used genetic markers are mutations in two genes related to DNA repair and recombination, namely *mutT2* codon 58 (G1286766C, *mutT2*-58) and *ogt* codon 12 (C1477596T, *ogt12*)^10^ as well as the insertion of IS6110 (insertion sequence 6110) in the NTF (noise transfer function) genomic region^8^. Other identifiers have also been used, such as a set of 44 SNPs (single nucleotide polymorphisms)^11^.

To understand their spreading potential, the geographical origin of L2 or the Beijing strains are sought and debated. A number of studies suggested that they started in Southern China^7,12^ and subsequently spread throughout Eastern Asia, from Tibet^13^ to Japan^14,15^. The Modern Beijing strains appeared to successfully co-expand with Han Chinese in Northern China^7^. It is unclear however whether the Modern Beijing strains originated there or in Southern China, moved with people and subsequently expanded in Northern China^7^. An alternate hypothesis is that the Beijing strains originated in Northern China^16,17^ as the Ancestral strains are common in Japan^14,15^ and Korea^18^ as well as in Jilin province in Northern China^12^.

Here we report the WGS analysis of *M*. *tuberculosis* isolates from a cohort of 1170 patients in Chiangrai province, Northern Thailand during 2003–2010. Some lineages and sublineages exhibited clear association with particular ethnicity of patients. Among the L2 strains, the Ancestral Beijing strains were associated with ethnic minorities. We discovered 40 isolates, which had only the *mutT2-58* mutation, considered as typical of the Modern Beijing strains. This sublineage was more common among Akha and Lahu ethnic minorities than Thai patients. Both tribes speak the Lolo-Burmese division of the Tibeto-Burman language family^19^. They were originally from Southern China and moved to northern Thailand only in the last 100 years^20^. We hypothesize that this sublineage descended from an ancestor that was an intermediate between the Ancestral and the Modern Beijing strains and, therefore, suggested that the evolutionary transition from the former to the latter occurred in Southern China.

## Results

### Phylogenetic analysis of *M*. *tuberculosis* in Chiangrai

**Figure 1:**
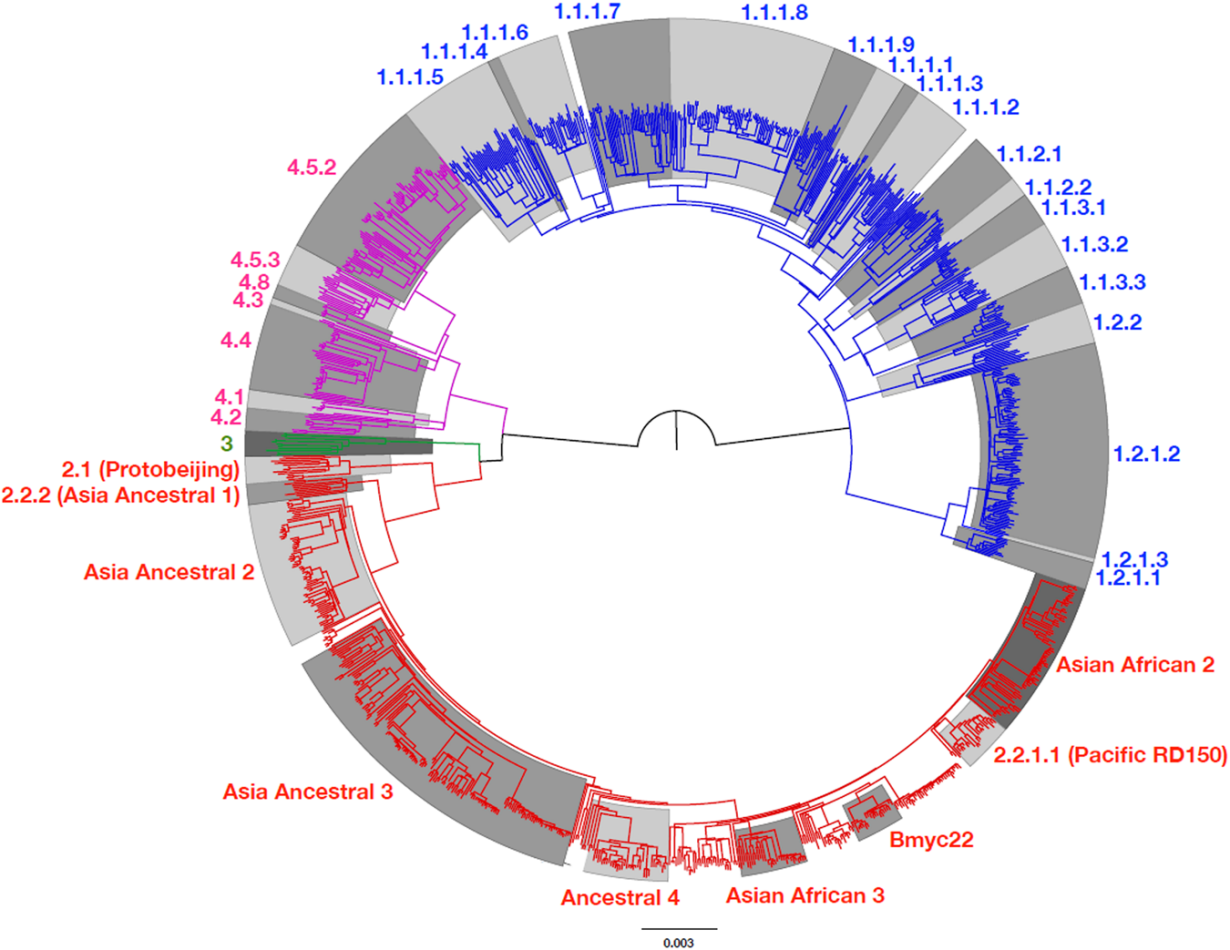
A phylogenetic tree of 1170 *M*. *tuberculosis* isolates from Chiangrai, constructed by using the Bayesian inference method. The shaded areas illustrate isolates belonging to the labelled sublineages. L1-4 are labelled with blue, red, green and purple lines, respectively. All L2 isolates, apart from 2.1 and L2.2.2 belong to L2.2.1. All isolates to the right of Ancestral 4 belonged to the Modern sublineages.

1170 successfully sequenced *M*. *tuberculosis* isolates were classified by phylogeny into Lineage 1–4 (L1-4) based on phylogenetic analysis of SNVs (single nucleotide variants) identified across their entire genomes. The results were completely consistent with the classification by experimental LSP (large sequence polymorphism). In total, 70937 SNVs were identified, with 33527 SNVs (47%) present in only a single isolate. The Bayesian and maximum likelihood phylogenies are shown in Figs 1 and S1 respectively. The number of isolates in each lineage was 480 (41.0%) for L1, 521 (44.5%) for L2, 11 (0.94%) for L3 and 158 (13.5%) for L4. The number of isolates belonging to each sublineage as well as some of their genetic descriptions are shown in Supplementary Table S1.

The classification of L1 isolates was previously reported^3^. The isolates belonging to L2 were classified based on the phylogenies shown in Figs 1 and S1A–C, and designated using the schemes proposed by Shitikov *et al*.^21^ and Mestre *et al*.^10^ sequentially. The details of the classification criteria are shown in Supplementary Table S1.

The prevalence of L2.1 was 2.3% of L2 isolates, which was similar to the prevalence reported from Chiba prefecture in Japan (2.35%)^22^ but lower than that reported from Guangxi (6.1%)^12^, consistent with the hypothesis that L2 originated in Southern China^7,12^.

The other 509 L2 isolates belonged to L2.2, which is congruent with the Beijing family, with 9 belonging to L2.2.2 or Asia Ancestral 1. The remaining 500 all belonged to L2.2.1.

235 (47%) of L2.2.1 belonged to the Modern Beijing sublineages, indicated by the presence of both C1477596T (*ogt12*) mutation and a copy of IS6110 in the NTF region. They also had 41 out of the previously reported 44 specific SNPs of the Modern Beijing sublineages^11^, as shown in Supplementary Table S2B. The other 265 isolates of L2.2.1, therefore, belonged to the Ancestral sublineages. Unlike in Bangkok in Central Thailand^23^ and many other places, there were more Ancestral than Modern Beijing isolates in Chiangrai.

There were 5 subsets of Ancestral Beijing strains belonging to L2.2.1 as shown in Supplementary Table S1. The majority (64 and 164 isolates) belonged to the Asia Ancestral 2 and 3 sublineages respectively. Nine and six isolates fit the descriptions of Bmyc6 and Bmyc26 respectively^21^. Each of them was not monophyletic, however, and were therefore considered as unclassified. The last sublineage, comprising 40 isolates, unexpectedly had one of the barcoding SNPs of the Modern sublineages, G1286766C (*mutT2* codon 58) but not the *ogt12* mutation. This sublineage did not have the 44 SNPs proposed to be specific to the Modern Beijing strains^11^ and most of them did not have IS6110 in the NTF region either. Five of the isolates had a copy of IS6110 inserted in the NTF region, at the position 3493756 and in the opposite orientation with respect to the one typically found in the Modern Beijing strains. This new sublineage, designated as L2.2.1.Ancestral 4, had a relative small average intragroup pairwise SNV distance of 127.4 and a high fixation index of 0.629, as shown in Supplementary Table S1, as well as a clear separation from other Ancestral Beijing strains in a principal component analysis (PCA) plot shown in Supplementary Fig. S2C. A single isolate with only *mutT2-58* mutation was previously reported from a patient in Shanghai^11^. As shown in Fig. 1, 2.2.1.Ancestral 4 appeared most closely related to the Modern Beijing strains among all Ancestral Beijing strains. Its average pairwise SNV distance from the Modern Beijing strains (286.7) was even smaller than that from other Ancestral Beijing strains (325.1). Hence, although 2.2.1.Ancestral 4 fit the definition of the Ancestral Beijing strains, it was genetically closer to the Modern Beijing strains and probably descended from an ancestor that was on an evolutionary transitional path from the Ancestral to the Modern Beijing strains.

In order to confirm that L2.2.1.Ancestral 4 was not previously underreported due to misclassification, we reinvestigated the reported WGS of 279 L2 isolates from China^6,7,16,24,25^. Only a single isolate from Fujian^24^ was identified as belonging to L2.2.1.Ancestral 4. A phylogenetic tree of the isolate together with other L2.2.1.Ancestral 4 isolates reported in this study is shown in Supplementary Fig. S3, which reveals that the Fujian isolate was closer to the root of tree and thus suggests that L2.2.1.Ancestral 4 in Chiangrai diverged after the isolate.

The 235 Modern Beijing isolates could be identified into six major groups. The numbers of isolates belonging to the L2.2.1.1 (Pacific RD150) and the Asian African 3 sublineage were 22 (9.3% of Modern Beijing strains) and 29 (12%) respectively. 70 (29.5%) isolates had the SNPs A2376135G and G2532616A, indicating that they belonged to either the Asian African 2 sublineage or L2.2.1.2 (Asian African 2/RD142) with only two of them having the RD142 deletion and, therefore, belonging to L2.2.1.2. 21 isolates (8.9%) had a mutation, C720902T, previously described as a marker specific to a single isolate from Thailand, designated as Bmyc22^10^. Unlike the cases of Bmyc6 and Bmyc26, all Bmyc22 isolates were monophyletic. A single isolate could be classified as Bmyc20^10^. The other 92 Modern strains did not fit to any previously described subsets of L2.2.1 and were referred to here as unclassified Modern Beijing isolates.

There were only 11 L3 isolates in this study. All cannot be subclassified based on Coll’s scheme^26^. 158 L4 isolates diversified into three major branches as shown in Supplementary Fig. S1D–E, which could be further classified^26^ into 6 sublineages, corresponding to 4.1–4.5 and L4.8. The majority of the L4 isolates belonged to L4.5 (93 isolates, 58.9%) and L4.4.2 (37 isolates, 23.4%). Both were similarly found in high frequencies in China^27^. The L4.5 strains did not include any isolates with SIT127 or related strains, which were recently described as L4.5.1/Iran^28^. L4.5 strains were tentatively classified into two subgroups, namely L4.5.2 and L4.5.3, based on their relatively large between group mean pairwise SNV distances (391.1) and fixation indices of 0.304 and 0.268 respectively, as well as the PCA plot shown in Supplementary Fig. S2D.

### Spoligotypes

The spoligotypes of 480 L1 isolates were previously described^3^. The spoligotypes of the L2-4 isolates are shown in Supplementary Table S3A–C respectively.

As generally reported^29,30^, the most common spoligotype among L2 isolates (87.3%) were SIT1 (000000000003771) which was found in all sublineages except L2.1. Most spoligotypes were found in several sublineages, except 000000000000771 (SIT269), which were found only in L2.2.1.Asia Ancestral 3. There were 17 isolates with SIT523 (777777777777771), which was usually considered as a marker of L2.1 or the proto-Beijing sublineage. Indeed, 10 (83%) of the L2.1 isolates had the spoligotype while the others had unclassified spoligotypes of SIT1149 (777777777777331) and SIT1487 (000000007777731). However, the other SIT523 isolates belonged to L2.2.1.Asia Ancestral 2 and L1.1.1.2^3^, even though they were confirmed to be L2.2.1 and L1 respectively by LSP.

The most common L4 spoligotypes (46.2%) were 777777777760771 (SIT53), found in four of the six sublineages. All other spoligotypes of L4 could be derived from SIT53 by deletion, suggesting that the most recent common ancestor (MRCA) of L4 had the spoligotype of SIT53. Spoligotypic clades of L4 were not congruent with the SNP phylogeny either. Nevertheless, T2 clade belonged to L4.4 or L4.2 but not L4.5. T1 clade belonged mostly to either L4.4 and L4.5.2 while H3 clade were mostly members of L4.5.3.

### Clinical profiles

The lineages and sublineages correlated with patient ages, ethnicity, HIV infection status, and drug resistance phenotype as shown partially in Table 1 and in detail in Supplementary Table S4.

**Table 1:** The selected demographic and clinical profiles of four lineages and some selected sublineages of L2 and L4.

	L1	L2	*L2*.*2*.*1*/*Ancestral*	Asia Ancestral 2	Asia Ancestral 3	Ancestral 4	*L2*.*2*.*1*/*Modern*	L2.2.1.1 (pacific rd150)	Asian African 2	Asian African 3	Bmyc22	Unclassified Modern	L3	L4	L4.4	L4.5.2	L4.5.3	TOTAL
TOTAL patients	480	521	265	64	146	40	235	22	68	29	21	93	11	158	38	74	19	1170
Sex: Male	354	357	176	42	103	24	166	16	47	21	17	63	9	110	23	49	18	830
Female	126	164	89	22	43	16	69	6	21	8	4	30	2	48	15	25	1	340
Average Ages in years (SD)	51.1 (16.7)	42.3 (15.9)	*40*.*9 (15*.*7)*	39.2 (16)	41.9 (16.6)	40.6 (12.2)	*43*.*6 (16*.*1)*	44 (16)	43.1 (14.9)	44.2 (15.6)	33.9 (10.8)	43.3 (16.5)	41.4 (15.5)	42.2 (15.2)	42.8 (16)	40.6 (15.3)	46.7 (16.4)	45.9 (16.7)
Median Age in years	50	41	39	37.5	40	39	42	42	41.5	46	31	43	34	41	45	39)	44	45
(IQR)	(38–65)	(30–52)	*(29–51)*	(27–52)	(29–52)	(32–50)	*(30–54)*	(33–52)	(31–53)	30–51	28–43	(30–53)	(29–49)	(32–52)	(33–54)	(29–52)	(32–52)	(33–58)
%with ages > 49	51.3	31.5	*30*.*2*	31.3	30.1	32.5	*31*.*5*	27.3	27.9	31.0	9.5	50.5	18.2	29.1	28.9	31.1	36.8	39.1
Thai patients	401	282	113	25	70	12	155	21	44	20	11	57	4	66	22	18	10	753
**RR by Ethnicity**																		
Other Tai ethnic groups	0.49 (0.32–0.77)	1.41 (1.08–1.83)	*2*.*46* *(1*.*68–3*.*59)*	3.17 (1.36–7.41)	1.89 (1.03–3.46)	1.10	*0*.*77*	0	1.20	0	1.20	0.93	3.30	2.20 (1.23–3.93)	0.60	3.67 (1.41–9.52)	3.96 (1.12–14.0)	57
Chinese	0.00	2.00 (1.43–2.81)	*2*.*78* *(1*.*39–5*.*54)*	2.51	2.69	0.00	*1*.*62*	0	2.85	0	5.70	1.10	0	2.85 (1.04–7.81)	2.85	3.49	6.28	12
Burman	0.31 (0.14–0.70)	1.87 (1.45–2.40)	*3*.*11* *(2*.*05–4*.*73)*	5.02 (2.07–17.1)	2.51 (1.26–4.98)	0	*0*.*97*	0	1.14	0	2.28	1.32	12.55 (2.39–65.9)	0.76	0	1.39	2.51	30
Akha	0.29 (0.20–0.43)	1.50 (1.26–1.77)	*2*.*30* *(1*.*74–3*.*04)*	1.84	1.89 (1.25–2.86)	6.36 (3.04–13.31)	*0*.*99*	0	0.81	1.02	1.86	1.35	1.28	3.16 (2.23–4.47)	2.10	8.25 (4.71–14.46)	0	148
Lahu	0.18 (0.09–0.36)	1.67 (1.37–2.04)	*2*.*58* *(1*.*85–3*.*59)*	4.02 (2.01–8.04)	1.72	5.02 (1.94–12.99)	*1*.*10*	0	1.14	0.50	0.91	1.76	2.51	3.04 (1.96–4.73)	1.37	6.69 (3.35–13.36)	3.01	75
Other Tibeto-Burman family	0.54	1.91 (1.18–3.07)	*3*.*81* *(1*.*96–7*.*39)*	4.30	3.07	8.96 (1.34–59.90)	*0*.*69*	0	0	0	0	1.89	0	0	0	0	0	7
Hmong-Mien	0.35 (0.13–0.98)	1.50	*2*.*92* *(1*.*63–5*.*11)*	1.88	2.69 (1.12–6.47)	7.84 (1.91–32.21)	*0*.*30*	0	0	2.35	0	0	11.77 (1.39–99.47)	2.14	0	5.23 (1.32–20.67)	4.71	16
Other ethnic minorities	0.45 (0.21–0.96)	1.40	*2*.*54* *(1*.*43–4*.*49)*	5.74 (2.19–15.02)	1.54	2.99	*0*.*69*	0	1.63	0	0	0.63	0	2.71 (1.22–6.04)	1.63	3.98	0	21
%with HIV	20.6	14.9	*14*.*4*	14.3	15.9	7.5	*15*.*5*	14.3	16.7	13.8	38.1	10.8	10	14.0	16.2	6.8	21.1	17.3
**%Resistance**																		
RMP	2.7	6.3	*4*.*5*	1.6	4.6	0.0	*7*.*0*	5.3	12.1	21.4	0	1.1	20.0	4.6	5.4	6.9	0	4.8
INH	9.5	16.2	*8*.*3*	8.2	6.9	5.7	*23*.*3*	15.8	24.2	39.3	4.8	24.2	50.0	13.7	16.2	8.3	15.8	13.5
STM	3.4	15.1	*7*.*9*	11.5	6.2	8.3	*22*.*9*	15.8	21.2	57.1	0	19.8	20.0	7.2	21.6	2.8	5.3	9.3
EMB	0.5	2.5	*1*.*2*	1.6	0.8	0	*3*.*5*	10.5	4.5	10.7	0	0	10.0	2.0	2.7	2.8	0	1.6
% MDR	2.27	4.70	*2*.*07*	0	1.54	0	*6*.*61*	5.26	12.1	17.9	0	1.10	20.0	3.92	5.41	5.56	0	3.75

More detailed information is in Supplementary Table S4. SD: standard deviation, IQR: interquartile range, RR: risk ratios, HIV: Human Immunodeficiency Virus, RMP: rifampin, INH: isoniazid, STM: streptomycin, EMB: ethambutol, MDR: resistance to both RMP and INH. Columns displaying the information for the Ancestral and Modern Beijing groups of L2.2.1 are with italized numbers.

The average age of all patients was 45.9 ± 16.7 years while the average ages were 51.1 ± 16.7 years for L1, 42.3 ± 15.9 years for L2, 41.4 ± 15.5 years for L3 and 42.2 ± 15.2 years for L4. Among L2, patients infected by L2.1 was the oldest and patients infected by Modern Beijing strains were slightly older than those infected by the Ancestral Beijing strains. The average age of patients infected with L2.2.1.Bmyc22 was the lowest among Modern Beijing sublineages. 1117 patients identified their own ethnicity. 753 (67%, 753/1117) were Thais and 57 patients identified themselves as Lao or other ethnic groups who speak the Tai-Kadai language family. 259 (23%, 259/1117) patients belonged to Tibeto-Burman speaking tribes, including 30 Burman, 148 Akha, 75 Lahu and 7 others, including Karen and Lisu. The numbers of other ethnicities were 12 for Chinese, 16 for Hmong-Mien and 21 for other ethnic minorities. Many of the ethnic minority people reside mostly in the mountainous areas and are generally called collectively as hill tribes.

**Figure 2:**
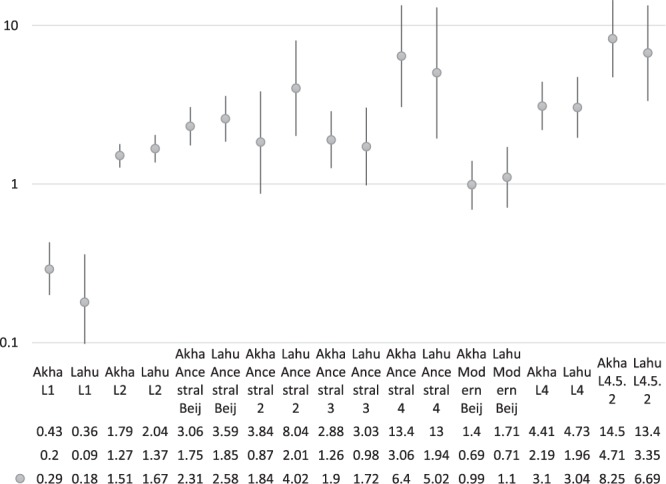
The risk ratios, of which the mean values are presented by grey dots with the lines showing 95% confidence intervals, of Akha and Lahu tribes compared to Thais for infections with some selected lineages and sublineages of *M*. *tuberculosis*. The Y axis is on a logarithmic scale. The vertical lines that intersect the horizontal line 1 indicate statistically non-significant risk-ratios. The risk ratios for L1 were much lower than one while the reverse was true for L2 and L4. The highest risk ratios were observed for L2.2.1.Ancestral 4 and L4.5.2.

The lineage frequency distributions were significantly different between Thais and non-Thai (p < 2.2 × 10^−16^, χ^2^_(3)_ = 139.68) as shown in Table 1 and Supplementary Table S4. The L1 lineage, particularly L1.1.1 (p = 7.911 × 10^−13^, χ^2^_(1)_ = 51.30), L1.2.1 (p = 9.719 × 10^−10^, χ^2^_(1)_ = 37.38) and L1.2.2 (p = 0.029, χ^2^_(1)_ = 4.80), was significantly more common among Thais while the L2 (p = 8.803 × 10^−11^, χ^2^_(1)_ = 42.07) and L4 (p = 3.287 × 10^−10^, χ^2^_(1)_ = 39.496) lineages were significantly more common among non-Thai patients. The higher prevalence among non-Thai individuals was found only for the L2.2.1.Ancestral sublineages but not for the L2.2.1.Modern sublineages as indicated by the risk ratios (RR) in Supplementary Table S4. Most patients infected with L2.1 were Thai, but the number of samples were small. Among specific ethnic minority groups, the distributions of sublineages among the linguistically and genetically related Akha and Lahu, were similar but there were differential associations with L2.2.1.Asia Ancestral 2 and 3 sublineages. The RRs of both tribes were similarly highest, being > 5.0, for both L2.2.1.Ancestral 4 and L4.5.2, as shown in Fig. 2. The RRs of the small number of Hmong-Mien speaking patients were also high. The RRs were not significantly high for L4.5.3, supporting the separation of L4.5 into two sublineages.

HIV infection was significantly more common among patients infected with L1 isolates (99/480, 20.6%, p = 0.005842, χ^2^_(1)_ = 7.5985). Nevertheless, the infection rates generally varied with sublineages. The co-infection rate was highest for the Bmyc22 sublineage (8/21, 38.1%) among the Modern Beijing sublineages (p = 0.02391, χ^2^_(1)_ = 5.1014).

The rates of resistance to isoniazid (INH), rifampin (RMP) and streptomycin (STM) were significantly associated with lineages (p = 0.0001358 χ^2^_(3)_ = 20.468, p = 0.007583 χ^2^_(3)_ = 11.942, p = 8.687 × 10^−9^ χ^2^_(3)_ = 40.418 respectively). For all drugs, the resistance rates of L1 were significantly less than L2 isolates (p = 0.001406 χ^2^_(1)_ = 10.197 for INH, 0.01085 χ^2^_(1)_ = 6.4904 for RMP, p = 2.842 × 10^−8^ χ^2^_(1)_ = 30.813 for STM, p = 0.01833 χ^2^_(1)_ = 5.5642 for ethambutol (EMB) and p = 0.04106 χ^2^_(1)_. = 4.1734 for both INH and RMP respectively).

The modern Beijing strains were significantly more resistant to INH and STM than the Ancestral Beijing strains (p = 3.914 × 10^−6^ χ^2^_(1)_ = 21.306 and 3.254 × 10^−6^ χ^2^_(1)_ = 21.661 respectively). There were significant variations of the drug resistance among sublineages of Modern Beijing strains to RMP (p = 0.002805, χ^2^_(5)_ = 18.115), STM (p = 6.597 × 10^−05^, χ^2^_(5)_ = 26.675) and both INH and RMP (p = 0.009964, χ^2^_(5)_ = 15.095) with the Asian African 3 sublineage having the highest rates of resistance to all drugs.

In general, the clinical profiles of patients infected with various sublineages of L1 were fairly similar. However, the patient profiles among sublineages of L2 varied significantly, in particular the Modern sublineages. For example, isolates belonging to L2.2.1.1 (Pacific RD150) were not found in any non-Thai patients. while all the other sublineages could be found as shown in Supplementary Table S4. The Asian African 3 sublineage had high drug resistance rates while the rates of Bmyc22 were very low. The patients infected by the latter tended to be young (average age = 33.9 ± 10.8 years) but had a high rate of HIV infection. The variations between sublineages may explain the variations in the results from previous phenotypic association studies of the Beijing strains^31^, which could be due to the differences in sublineage compositions.

### Geographical distribution of *M*. *tuberculosis* lineages and sublineages in Chiangrai

**Figure 3:**
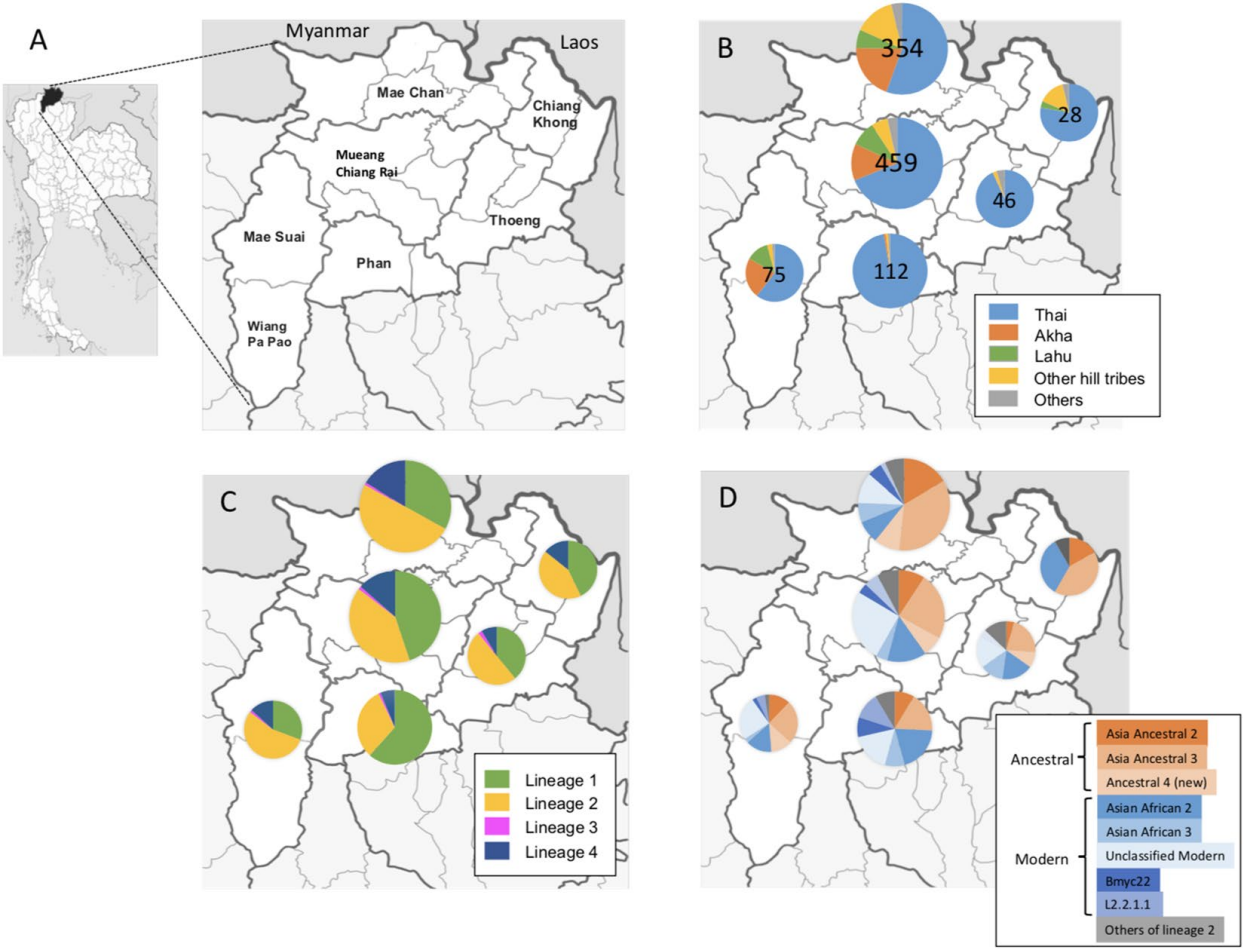
The geographical distribution of patient ethnic groups, various lineages and sublineages of L2 in Chiangrai. 1,098 patients with the geographic information were included. (**A**) Chiangrai is divided into administrative districts. We summarized the data for 6 main areas as shown on the map. (**B**) The distribution of patients in each area by ethnic groups. The number of tuberculosis patients in each area is shown in the pie charts. (**C**) The distribution of the four lineages in each area (N = 1,098). (**D**) The distribution of sublineages of L2 in each area. (N = 481).

The geographic distribution of various *M*. *tuberculosis* lineages and sublineages were studied by associating sublineage information to the addresses of the patients in various administrative districts in Chiangrai. Although there has been considerable mixing of residential areas of various tribes. Akha and Lahu populations reside predominantly in the mountainous North and West and the Central part of Chiangrai while Hmong-Mien tribes are mostly in the East and Northeast. The patients living in the river plains in the South, Central, and the East were predominantly Thais as shown in Fig. 3B. The map of the distribution of lineages revealed a large fraction of L2 isolates, and particularly the Ancestral Beijing strains, among patients in the North near Myanmar border as shown in Fig. 3C,D. L2.2.1.Ancestral 4 was common in the North, West and Central where Akha and Lahu mostly reside. A relatively large fraction of L4-infected patients also resided in the North and Central while L1 infected patients contributed to the largest fraction of the patients residing in the plain the South and Central part. The Ancestral Beijing strains was also found in a considerable proportion in the Northeast where Hmong-Mien minority commonly dwell as shown in Fig. 3D.

### Lineage-specific single nucleotide variants (LS-SNVs) of L2.2.1.Asian African 3

To explain the remarkable high drug resistance rate among L2.2.1.Asian African 3 strains, all 34 identified sublineage specific mutations were examined. 19 SNPs were non-synonymous, with 11 being in hypothetical proteins as shown in Supplementary Table S5. Mutations in 13 genes were predicted to affect their protein functions. An interesting function-affecting mutation was found in an essential gene, *aftD*, encoding arabinofuranosyltransferase enzyme. This enzyme is involved in the synthesis of the arabinan domain of major mycobacterial cell envelope glycolipids, arabinogalactan and lipoarabinomannan^32,33^. The SNP may affect cell wall functions and isoniazid activity^34^.

Other notable mutated genes include *uvrD1*, which plays a role in the repair of multiple forms of DNA damage, including site-specific chromosomal double-strand breaks. Ablation of UvrD1 functions sensitizes the bacteria to ionizing radiation^35,36^. However, the Asian African 3 specific SNP in *uvrD1* was not predicted to affect its function even though a minor effect could not be ruled out. UvrD1 plays a role in persistence of *M*. *tuberculosis* infection in a murine model^37,38^.

Other virulence-related genes that harbour function-affecting SNPs specific for the Asian African 3 strains include *fadD29* responsible for phenolic glycolipid (PGL-tb) biosynthesis^39^, *cstA*, carbon starvation protein A homolog, *mce1A*, related to bacterial cell entry to host cells^40^ and *Rv0140*, a reactivation-associated antigen^41^.

## Discussion

The population structure of *M*. *tuberculosis* in Chiangrai is highly complex, comprising L1-L4, and each lineage, except for L3, could be classified further into several sublineages. This might be results of the complex history of Chiangrai which has been a settlement since 7^th^ century and alternately controlled by several tribes now residing in Thailand, Myanmar and Lao. It is also settled by several hill tribes and more recently by Chinese immigrants.

This study revealed several interesting features of the population structure of *M*. *tuberculosis*, which have a number of implications.

First, Chiangrai was unusual in that the Ancestral Beijing strains were more common than the Modern Beijing strains, which was different from China in the north^27^ and Bangkok in the south^23^. The Ancestral Beijing strains were associated with ethnic minorities, who mostly migrated from Southern China through Northern Myanmar, Lao and Vietnam. These areas were, therefore, likely to have high prevalence of Ancestral Beijing strains as well.

We previously compared country-wide proportions of Beijing isolates and the isolates with single-banded IS6110 RFLP, majority of which belonging to Lineage 1. The proportion of Beijing strains was higher among younger patients and generally decreased with distances from Bangkok, the capital of Thailand, while the reverse was true for the single-banded isolates^9^. Lineage 1 was hypothesized to be endemic in Thailand before the emergence of the Beijing strains, presumably by various immigration waves of Han Chinese mostly via marine routes through Bangkok. The discovery of a large proportion of the Ancestral Beijing sublineages and their association with ethnic minorities who originally migrated from Southern China, indicates the significance of the land route in the movement of Ancestral Beijing sublineages as well.

Second, there were a considerable number of isolates genetically similar to rare or belonging to unknown genotypes, such as L2.2.1.Bmyc22^10^, the unclassified Modern Beijing sublineages and surprisingly L2.2.1.Ancestral 4. This study thus demonstrated the need for careful phylogenetic analysis in WGS studies of *M*. *tuberculosis*. The finding that different bacterial genotypes were associated with different ethnic groups suggested a possibility of encountering new or rare genotypes when bacteria circulating in a new population are to be studied.

The third interesting finding was the variations of demographic and clinical profiles among different sublineages, particularly among L2, which might reflect different activities of transmission. For example, the very low intragroup average pairwise SNV distance of the Bmyc22 (49.8), together with the low average age and high HIV co-infection rate suggested a relatively high recent transmission activity. This knowledge may be useful for developing public health control of the spreading sublineages. Differential drug resistance rates were also observed, especially among the Modern Beijing sublineages. The classification of Beijing strains only broadly into the Ancestral and the Modern strains may not be adequate for indepth genotype-phenotype association studies, as the results may change^11,31^ if the compositions of sublineages vary. With the increasing availability of WGS data of *M*. *tuberculosis* in the near future, an internationally agreed guideline for genotypic classification of *M*. *tuberculosis* is needed. The high resistance rates among some sublineages also highlight the need for drug susceptibility studies in controlling tuberculosis. It still needs to be investigated whether the drug resistance variability contributes to the difficulty in controlling tuberculosis in some areas or not.

The fourth finding is the discovery of L2.2.1.Ancestral 4 sublineage, which harbored both *mutT4-48* and *mutT2-58* but not *ogt12* mutations. This indicated that the identification of the Modern Beijing strains by barcoding SNPs of *mutT2-58* and *ogt12* mutations^10^, needs revision probably by including more SNPs from the set of 41 SNPs specific to the Modern strains. It also posed some doubts on the hypothesis that the *MutT2*−58 together with the *MutT4*-48 mutations might provide some evolutionary advantages of rapid adaptation for the Modern Beijing strains, by increasing their mutation rates, because both mutations were found in L2.2.1.Ancestral 4 and the *ogt12* mutation was silent^10,42^. The selective advantages may be explained by the 41 specific SNPs. Alternatively, the mutations in the putative mutator genes by themselves might decrease the fitness of the strains, which required compensatory mutations^42^ existing only in the Modern Beijing strains or the apparent advantages of the Modern Beijing strains may be actually contributed by some sublineages, and not the entire group of Modern sublineages. In any case, it was clear that *mutT2-58* could not be reliably used as a SNP marker for identifying the Modern Beijing strains.

The fact that a considerable number of L2.2.1.Ancestral 4 existed in Chiangrai and was much more prevalent among the two linguistically and genetically related Akha and Lahu tribes^43^ than the native Thai population illustrates the phylo-ethno-geography of *M*. *tuberculosis* in a local and regional scale. As Akha and Lahu were originally from Yunnan or Southwestern China^20^, the sublineage might be originally present there. An isolate in Fujian which appeared to branch out before the isolates in Chiangrai conformed to this hypothesis. The sublineage might have adapted and become endemic to Akha and Lahu but was subsequently out-competed by the Modern Beijing sublineages in most parts of China. Alternatively, both tribes, which dwell in overlapping areas, might have acquired the sublineage along the southward migratory route, such as in Eastern Myanmar. More information about the population structures of *M*. *tuberculosis* in Myanmar or Lao are required to further examine this hypothesis. Nevertheless, it is however unlikely that Akha and Lahu acquired and adapted to the sublineage in Thailand because they only recently arrived at Northern Thailand about 100 years ago. Moreover, at first they dwelled almost exclusively in the mountainous areas separated from other ethnic groups. It was only after 1980s with several development programs that they had become integrated economically and then socially with native Thais on the plain of Chiangrai. Recently there has also been some slow but continuous physical migration waves of some hill tribes, particularly Akha to urban areas of Chiangrai. We hypothesized that along with these events, L2.2.1. Ancestral4 and L4.5.2, which might be previously more exclusive among hill-tribes have been spilling over to Thais and other ethnic populations.

The possible origin of L2.2.1.Ancestral 4 in Southern China was supported by the high prevalence of L4.5.2 among Akha and Lahu as well. L4.5 was previously described as a specialist L4 sublineage^44^, due to its geographic restriction mainly only to China. The phylogeny suggested the separation of L4.5 into two subgroups, L4.5.2 and L4.5.3, which were distinct by their spoligotypes, drug resistance rates and the associations to Akha and Lahu. The associations were strong and significant only for L4.5.2. L4.5 has been proposed to have separated from other sublineages of L4 for at least a millennium and may have spread from Tibet^28^ to other places in China. This study suggested that L4.5.2 might have accompanied Tibeto-Burman tribes who migrated southward as well. In this case, the association of the sublineage with ethnicity in Chiangrai may be mainly due to the founder effect, although some contribution of co-evolution might also exist.

**Figure 4:**
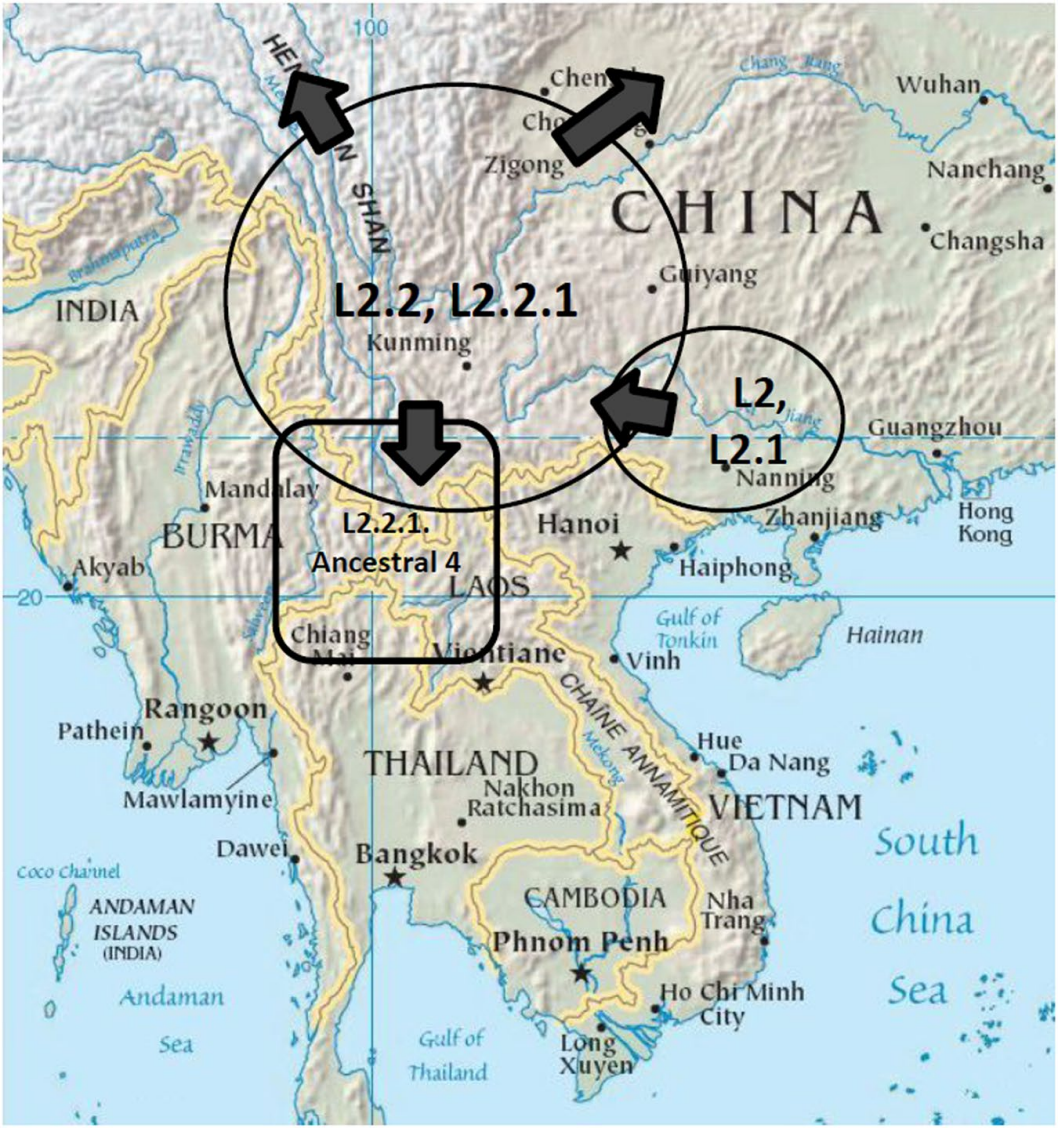
A map of the approximate area dwelled by Akha and Lahu^54^, represented by the rectangle, illustrating a hypothetical scenario of *M*. *tuberculosis* L2 evolution. The map was adapted from a public template (https://commons.wikimedia.org/wiki/File:Mainland_Southeast_Asia.png), using Google Slide (https://docs.google.com/presentation). As L2.1, the most basal sublineage of L2 was most common in Guangxi, the origin of L2 may be there. Subsequently, L2 strains might have spread to the mountainous areas on the west of Guangxi, and diversified to become L2.2 and L2.2.1/Ancestral strains. The strains circulated among various ethnic groups. The harsh terrain and alternate high mountains and valleys running mostly from North to South support a highly diverse species adapted to different latitudes and altitudes and allow societies with different cultures and languages to thrive, presumably with minimal genetic admixing for a prolonged period of time. This might have allowed for the diversification of L2.2.1, each of which co-evolved with a different ethnic group. Potentially facilitated by the Ancient Tea Horse Road or Southwest Silk Road^45^, some of the bacteria might have eventually been transmitted to a much larger and denser population of Han Chinese and further evolved to become the current Modern Beijing strains. Some of the minority tribes migrated to the South carrying some sublineages, such as L2.2.1.Ancestral 4 and L4.5.2, with them.

The presence of the Ancestral 4 sublineage in the ethnic groups originally from Southern China suggested that the mutations that transformed the Ancestral sublineages may have been acquired gradually before finally reaching the state of the Modern Beijing strains. Some of these changes occurred in Southern China probably in the areas historically occupied by Akha and Lahu ethnic groups. It should be noted that, currently, both tribes are scattered across a mountainous area, extending from the Himalayan mountains toward the South China Sea and covering Southern China, Northern Myanmar, Thailand, Laos and Vietnam as illustrated in Fig. 4. This area was also populated by numerous groups of other ethnic minorities or hill tribes, speaking several hundred different languages belonging to various language families, including the Tibeto-Burman, Tai-Kadai, Hmong-Mien and Austroasiatic. The area is composed of mountainous ranges that run mostly from the temperate north to the tropical south alternating with deep valleys containing river plains. The vast diversity of altitude and latitude create diverse climate and environments, allowing for vast biodiversity^45^, where various ethnic groups can thrive separately in different habitats. The high language diversity suggests a high genetic diversity among ethnic minority populations. The areas are remote due to harsh mountainous terrain, allowing many tribes to live in their traditional ways and probably maintain their genetic segregation. Many areas are not yet covered well with modern health services and hence not studied. It is possible that there are more variants of the Beijing strains, some of which may have partial characteristics of the modern strains, circulating in the areas. Each may have been maintained by co-evolution with specific ethnic groups.

This hypothesis, together with the high prevalence of L2.1 in Guangxi, conforms to the hypothesis that the origin of the Lineage 2, L2.1 and probably L2.2 or the Beijing strains was in Southern China^7^, where L2.2.2 or the RD181-positive Beijing strains were also highly prevalent^12^. The early L2.2 or Ancestral Beijing strains spread widely from Tibet^13^ to Japan^14,15^. In south Eastern Asia they may have diversified by co-evolving with various genetically diverse non-Han Chinese ethnic tribes in the segregated mountainous areas. The emerging sublineages fail or thrive for millennia, resulting in the present variety of the Ancestral Beijing lineages. Some of them presumably gained novel mutations, making them more and more similar to the present Modern sublineages. They might have been subsequently transmitted to the ancestors of Han Chinese, as illustrated in Fig. 4. As the latter population expanded in Northern China, the Modern Beijing sublineages also co-expanded to become predominant strains throughout China and East Asia as found presently^8^. Further studies in the area would provide more insight to the evolution of L2 or the Beijing strains, allow us to understand more about the evolution of pathogenesis of in these microbes and hopefully allow the design of a better tuberculosis control tool.

## Conclusion

WGS of *M*. *tuberculosis* isolates in Northern Thailand revealed an intriguing population structure, with the Ancestral Beijing strains being slightly more common than the Modern Beijing strains and the presence of several rare or new sublineages of the Beijing strains. The sublineages were differentially associated with the resistance patterns of the bacteria and the demographic and clinical profiles of the patients. The differential associations might explain the variations of the associations of the Beijing strains and phenotypes in different studies. A sublineage of the Ancestral Beijing strains had *MutT2-58* mutation, which is considered as specific to the Modern Beijing strains. The association of the sublineage with Akha and Lahu ethnic groups who migrated from Southern China to Thailand about 100 years ago, suggested the origin of the sublineage in Southern China. Consequentially, the evolutionary transition from Ancestral to Modern Beijing might be gradual and occur in Southern China. Understanding the molecular transitional path will provide better understanding of pathogenesis and help the rational development of various products, especially vaccines against tuberculosis.

## Methods

### Settings

This study included samples from chronic pulmonary tuberculosis patients registered in Chiangrai Province, Northern Thailand during 2003–2010. The isolates were collected as a part of a tuberculosis cohort study initiated by the Research Institute of Tuberculosis (RIT), Japan Anti-Tuberculosis Association (JATA), and Ministry of Public Health, Thailand^3^.

Chiangrai is an area with ancient settlement and has become a major tourist attraction site and a transportation hub to Myanmar, Lao and China through the Mekong River. The recent economic growth of Chiangrai has resulted in substantial migration of populations from other parts of Thailand as well as from neighboring countries. The population of Chiangrai is about 1.2 million with the tuberculosis incidence rate of 152.6/100,000 population in 2011. Most of the populations were native Thais who may be genetically admixed with Chinese and others. Ethnic minorities constitute a considerable proportion of Chiangrai populations. Most of them live in villages in mountainous areas and are known collectively as hill tribes^20^. There were six major tribes based on their spoken languages. Akha, Lahu, Karen and Lisu speaks languages belonging to the Tibeto-Burman family while Hmong and Iu-Mien (Yao) languages belong to the Hmong-Mien family^46^. The ethnicity of the patients was recorded as self-identified.

Akha and Lahu speak the Lolo-Burmese division of Tibeto-Burman language family^19^. They probably started to establish in Yunnan 2000 years ago from the Tibet region. They migrated through Myanmar and arrived at Northern Thailand about 100–200 years ago^20^.

### Ethics Statement

The project was approved by the Ethical Committees of Chiangrai Prachanukroh Hospital, Chiangrai and the Ministry of Public Health, Thailand. Informed consent was obtained from all participants and/or their legal guardians. All methods were performed in accordance with the relevant guidelines and regulations.

### Bacteria

Bacterial samples from 1187 patients were recultured in Lowenstein-Jensen medium in an appropriately contained clinical microbiology laboratory in Chiangrai using standard biosafety protocols and equipment. DNA was extracted as previously described^3^. All the processes were performed in Class II biosafety cabinets.

### Bacterial genotyping

LSP and spoligotyping of all isolates were experimentally determined as previously described^3^.

### Whole Genome Sequencing and SNV analysis

The samples were sequenced on the Illumina HiSeq 2000 platform to produce paired end reads, at the Sanger Institute, UK and processed as previously described^3^. The number of available reads with acceptable scores of five isolates were too small for further analysis. Twelve samples appeared to contain mixed nucleotide sequences and were also not further studied. The sequencing data of samples used in this paper were submitted to the European Nucleotide Archive (ENA) of EMBL-EBI which are mirrored in the Sequence Read Archive (SRA) database. Actual read sequences can be queried and downloaded directly from the SRA database using the study accession numbers ERP006738 and submission accession numbers ERA398050, ERA407418, ERA411689, ERA414376 and ERA428771.

### Phylogenetic analysis

The remaining SNVs from 1170 isolates were used for phylogenetic tree reconstruction with Maximum Likelihood (ML) and Bayesian Inference (BI) methods using PHYLIP^47^, PhyML^48^ and MrBayes^49^, respectively. The ML trees were supported by 1,000 replicates of pseudosamples, and the BI tree was supported with posterior probabilities. The tree was visualized by FigTree version 1.4.2. Principal Component Analysis (PCA) was done using Jalview 2.8.2^50^. The plots between the first three eigenvectors were examined.

Pairwise SNV distances were calculated using MEGA5^51^. Fixation index (F_ST_) is calculated based on the formula (π_between group_ – π_within group_)/π_between group_, where π_within group_ is the average pairwise SNV distance within a group and π_between group_ is the average pairwise SNV distance between all members in the group and all members not in the same group but in the same level of grouping. The statistical tests for the differences between π_within group_ and π_between group_ were done by Wilcoxon rank-sum test at α = 0.05.

### Analysis for IS6110 in the NTF region

The number and positions of IS6110 in the genome of each isolate was identified by ISMapper using the provided IS6110 sequence^52^ as query and H37Rv as the reference genome with default parameters.

### Classification of lineages and sublineages

The classification of L1 into sublineages was reported previously^3^. Classification of L2 was based on a scheme proposed by Shitikov^21^. Isolates that did not fit to any known sublineage were re-classified by the scheme of Mestre^10^. The details of the classification criteria are shown in Supplementary Table S1.

### Analysis of phenotypic data

The patient characteristics were described in descriptive statistics as presented in Table 1 and Supplementary Table S4. The associations between phenotypes and the four lineages were evaluated by one-tailed Pearson Chi-square test. The P-values less than 0.05 were considered as significant.

Associations with ethnicity were analyzed by categorizing patients based on self-identified ethnicity regardless of nationality. The risk ratio (RR) of each ethnic group was calculated compared to the Thai patients. The RR of all other Tai-Kadai speaking tribes including Lao were calculated together due to the small sample size. The RR for the Tibeto-Burman were presented for Akha and Lahu separately and all the others (which included Burman) combined. The Hmong-Mien group was composed of Hmong and Yao (Iu-Mien).

The HIV and drug resistance rates were calculated based on samples for which the data were available. The MDR rates were calculated based on the number of samples that both susceptibility to INH and RMP was known.

### Statistical analysis

Categorical variables of clinical profiles, such as sex, ethnicity, clinical presentation, HIV status, drug resistance were presented as number. Continuous variables were presented as the mean and standard deviation (SD) and the median and interquartile range (IQR). The chi-square test or Fisher’s exact test and Yates correction were applied in the analysis in this study as appropriate. A p-value of less than 0.05 was considered statistically significant. All statistical analyses were performed using R version 3.4.3 (R Foundation).

### Analysis for L2.2.1.Ancestral 4 in China

We investigated the presence of L2.2.1.Ancestral 4 in China based on the presence of *MutT2-58* but not *ogt12* mutations by analyzing the sequence deposited in the following accession numbers: SRA065095^24^, SRP051093^7^, and, ERP000111, ERP000124, ERP000192, ERP000276, ERP000436, ERP001731, ERP002617, ERP004677, ERP006989, ERP013054, SRA065095, SRP051093, and TB-ARC-Belarus^21^.

### Analysis of non-synonymous L2.2.1.Asian African 3 sublineage specific SNPs (LS-SNPs)

SNPs specific to L2.2.1.Asian African 3 were identified with the non-synonymous ones listed in Supplementary Table S5. The effects of the nonsynonymous LS-SNPs on protein function were predicted by two algorithms, Polyphen-1 and SIFT, from online consensus classifier PredictSNP1.0^53^. The nsSNPs influencing protein function were identified in 7 functional categories.

## Supplementary information

Supplementary Informaion for the manuscript

Supplementary Table S2

## Data Availability

The datasets used and/or analysed during the current study are available from the corresponding author on reasonable request.
