# Supplementary material for: A novel Ancestral Beijing sublineage of Mycobacterium tuberculosis suggests the transition site to Modern Beijing sublineages
Source: Sci Rep. 2019 Sep 23;9:13718. doi: 10.1038/s41598-019-50078-3 (PMC6757101; doi:10.1038/s41598-019-50078-3)
Supplement: Supplementary file 1 — Supplementary Informaion for the manuscript [file 41598_2019_50078_MOESM1_ESM.docx]

**Supplementary Information for the manuscript**

**A novel Ancestral Beijing sublineage of *Mycobacterium tuberculosis* suggests the transition site to Modern Beijing sublineages.**

Pravech Ajawatanawong^1^, Hideki Yanai^2^, Nat Smittipat^3^, Areeya Disratthakit^4^, Norio Yamada^5^, Reiko Miyahara^6^, Supalert Nedsuwan^7^, Worarat Imasanguan^7^, Pacharee Kantipong^7^, Boonchai Chaiyasirinroje^8^, Jiraporn Wongyai^8^, Supada Plitphonganphim^9^, Pornpen Tantivitayakul^10^, Jody Phelan^11^, Julian Parkhill^12^, Taane G. Clark^11^, Martin L. Hibberd^11^, Wuthiwat Ruangchai^1^, Panawun Palittapongarnpim^1^, Tada Juthayothin^3^, Yuttapong Thawornwattana^1^, Wasna Viratyosin^3^, Sissades Tongsima^3^, Surakameth Mahasirimongkol^4^, Katsushi Tokunaga^6^, Prasit Palittapongarnpim^1^ ^3^ *.

This supplementary PDF file includes the following information:

Supplementary Figures S1A-E, 2A-D and S3

Supplementary Table S1, 2, 3A-C, 4 and 5

References

Note: Supplementary Table S2 is provided as a separate excel files.


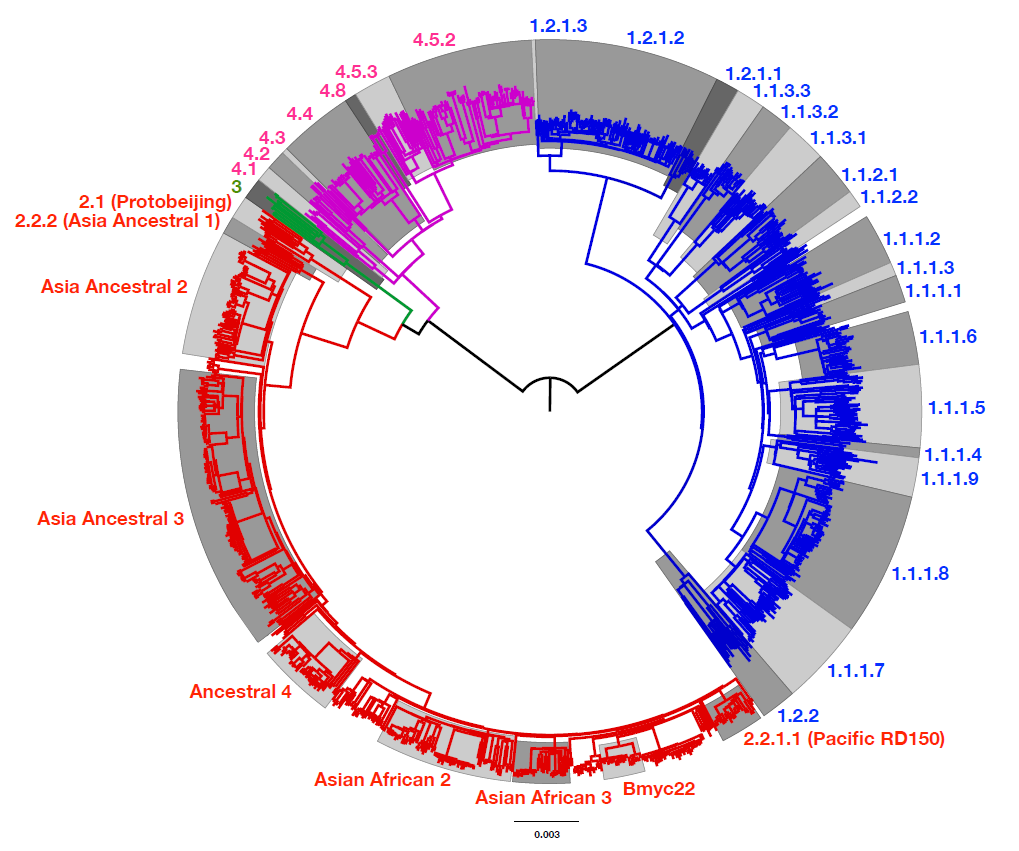


Fig S1A A phylogenetic tree of 1170 *M. tuberculosis* isolates from Chiangrai, constructed by the Maximum Likelihood method. Each shaded area illustrates isolates belonging to the labelled sublineage. L1-4 was labelled in blue, red, green and purple lines. Most of the L2 isolates belonged to L2.2.1. Classification of isolates into sublineages in this tree is congruent with the Bayesian inference tree shown in Fig 1, although the topological relationship between sublineages may be different.


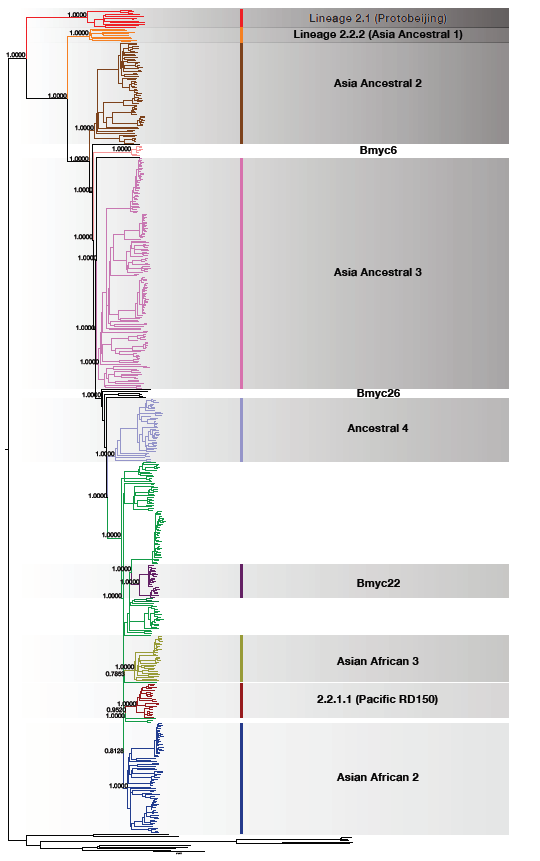


Fig S1B The phylogenetic tree of 521 L2 *M. tuberculosis* isolates from Chiangrai, constructed by the Bayesian inference method. All the isolates below the Ancestral 4 sublineage were the Modern Beijing strains. The unlabeled isolates were unclassified Modern Beijing isolates.


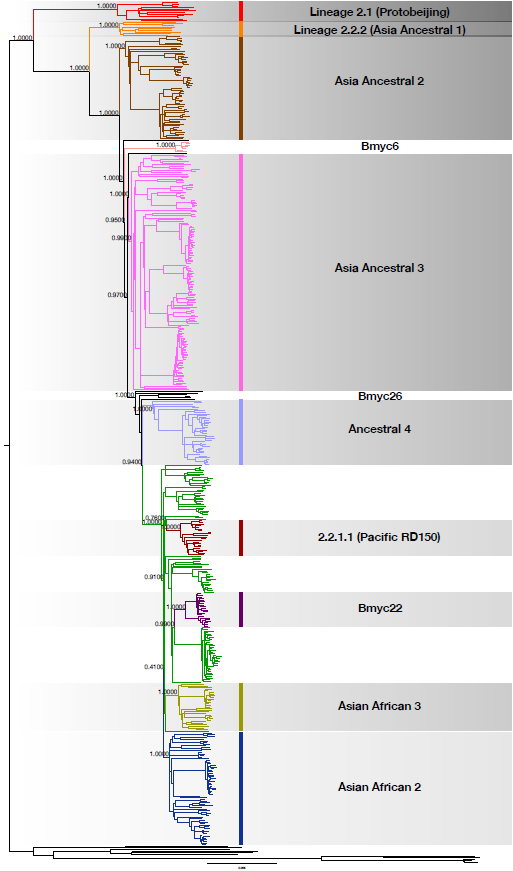


Fig S1C The phylogenetic tree of 521 L2 *M. tuberculosis* isolates from Chiangrai, constructed by the Maximum Likelihood method. All the isolates below the Ancestral 4 sublineage were Modern Beijing strains. The unlabeled isolates were unclassified Modern Beijing isolates. The cascading profile of the Ancestral sublineages is the same as Fig S1B. However, the topological relationship of the Modern sublineages were different.


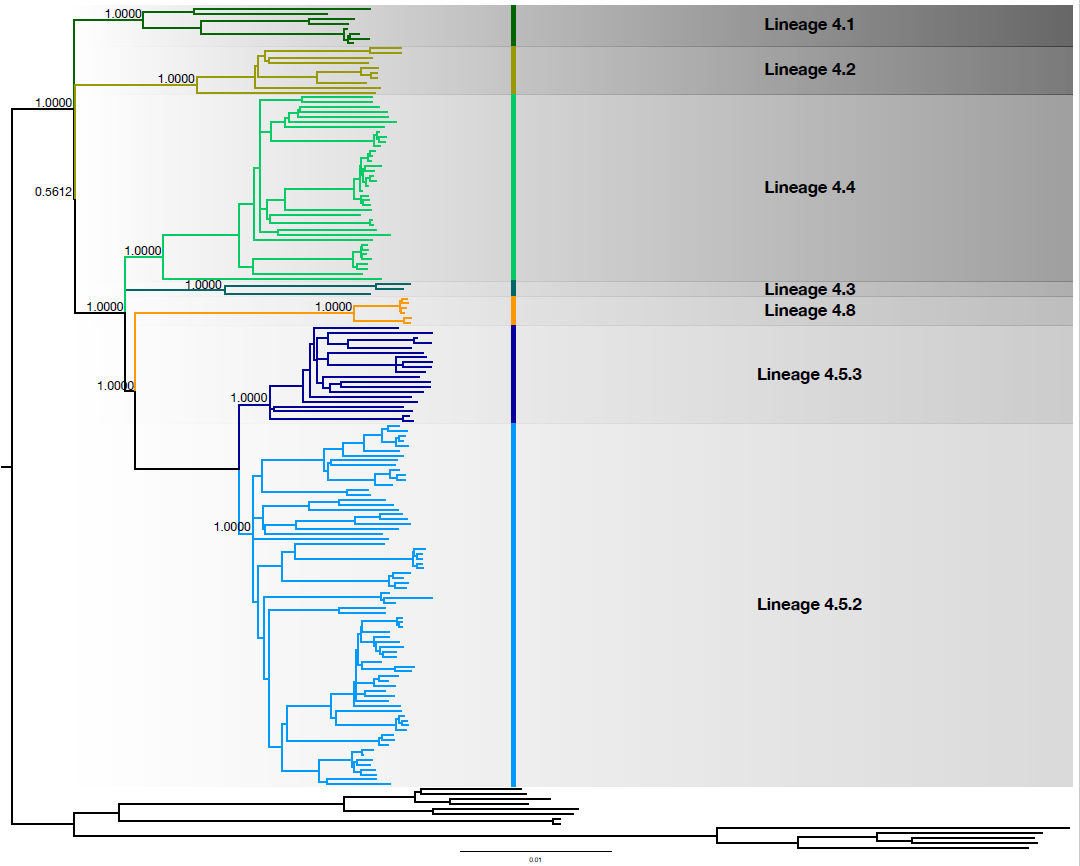


**Fig S1D** The phylogenetic tree of 158 L4 *M. tuberculosis* isolates from Chiangrai, constructed by the Bayesian inference method. Classification of isolates into sublineages is congruent with the maximum likelihood tree shown in **Fig S1E**. So are the topological relationships between sublineages.


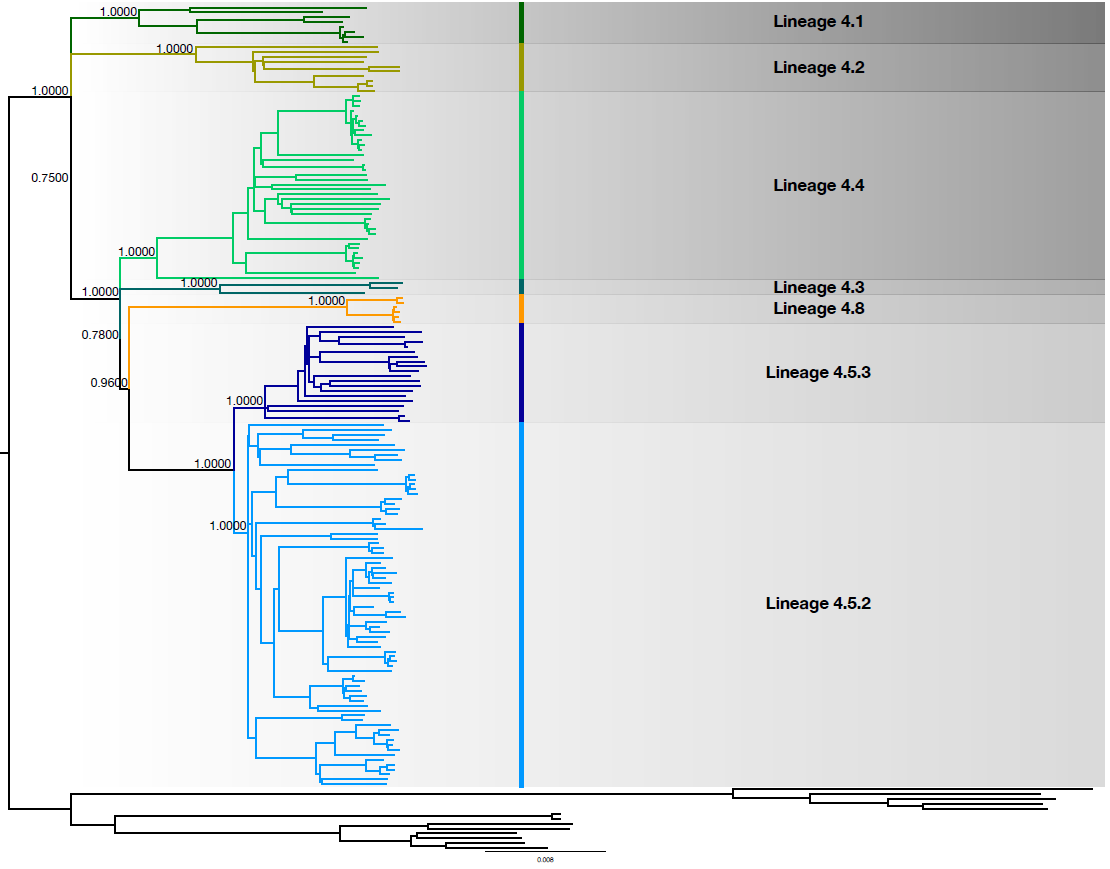


Fig S1E The phylogenetic tree of 158 L4 *M. tuberculosis* isolates from Chiangrai, constructed by the Maximum Likelihood method.


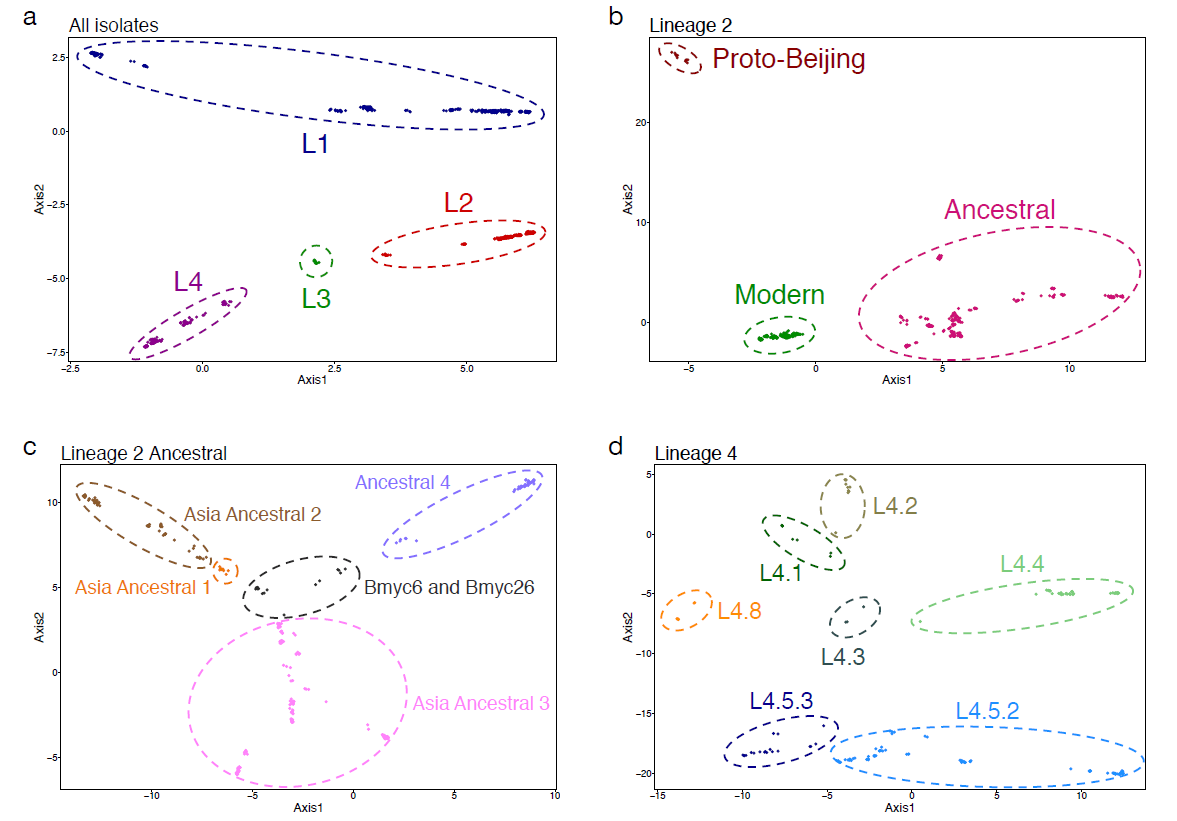


**Supplementary Fig S2** Principal component analysis plots for *M. tuberculosis* isolates in this study based on SNP. A. The plot for isolates belonging to all four lineages in this study. B. The plot for isolates belonging to L2. C. The plot for Ancestral Beijing isolates. D. The plot for the L4 isolates.

**
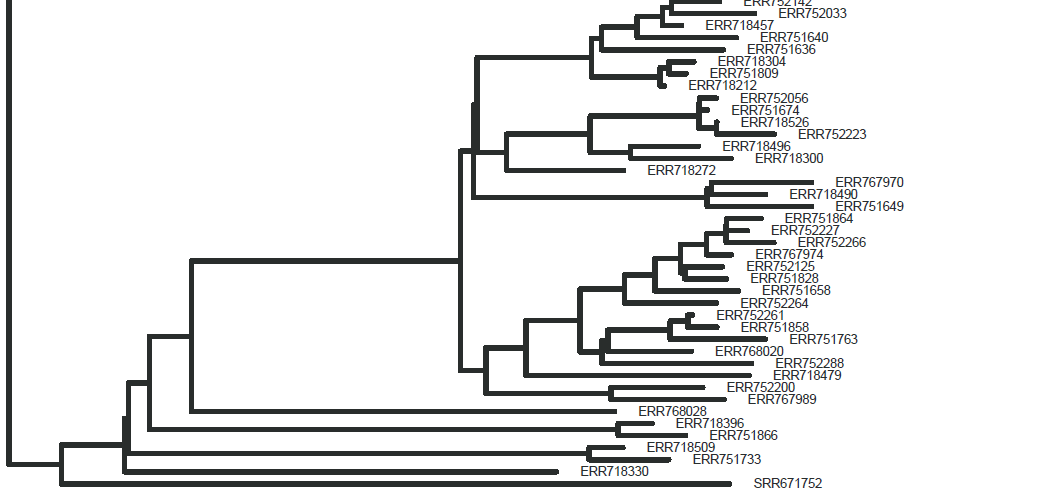
**

**Supplementary Fig S3** A Maximum Likelihood phylogenetic tree of all L2.2.1.Ancestral 4 isolates in Chiangrai together with the isolate from Fujian^1^ with the same mutation in *mutT2* and *ogt*. The Fujian isolate was the lowest branch in the picture.

**Supplementary Table S1** Summary of findings for each sublineage in this study. The information regarding L1 was previously published^2^. Specific SNPs appeared only in all members of the sublineages. The barcoding SNPs accorded to studies by Palittapongarnpim^2^, Shitikov^3^, Mestre^4^ and Coll^5^. The probability denotes the probability that the mean SNV distances of isolates in a group were different from those of other isolates that were not in the same group but in the same level of grouping, by the Wilcoxon rank-sum test. 105e: extended form of RD105.

| **SNP Lineage/ Sublineage (Equivalent names)** | **Number of isolates** | **Number of specific**  **SNVs** | **Coll’s classifi-cation** | **Specific RD** | **SNPs in DNA repair genes** | **Specific**  **barcoding SNPs^a^ in this study** | **Mean SNV distances within/**  **/between sublineages** | **Probability** | **Fixa-tion Indi-ces** |
| --- | --- | --- | --- | --- | --- | --- | --- | --- | --- |
| **1** | 480 | 550 | **1** | 239 |  | 615938 G/A |  |  |  |
| **1.1** | 354 | 29 | **1.1** | 239 |  | 4404247 G/A | 570.0/808.4.1 | <2.2x10^-16^ | 0.295 |
| **1.1.1** | 269 | 47 | **1.1.1** | 239 |  | 3021283 G/A | 451.1/749.7 | <2.2x10^-16^ | 0.398 |
| **1.1.1.1** | 14 | 57 | **1.1.1.1** | 239 |  | 3216553 G/A | 252.9/468.3 | 7.45x10^-6^ | 0.460 |
| **1.1.1.2** | 26 | 58 |  | 239 |  |  | 340.5/554.2 | 6.53x10^-10^ | 0.386 |
| **1.1.1.3** | 7 | 7 |  | 239 |  |  | 431.8/503.7 | 0.002331 | 0.143 |
| **1.1.1.4** | 5 | 56 |  | 239 |  |  | 224.2/477.2 | 0.01167 | 0.530 |
| **1.1.1.5** | 42 | 56 |  | 239 |  |  | 323.9/505.5 | 3.12x10^-15^ | 0.359 |
| **1.1.1.6** | 27 | 122 |  | 239 |  |  | 135.3/490.6 | 3.02x10^-10^ | 0.724 |
| **1.1.1.7** | 44 | 38 |  | 239 |  |  | 269.8/453.4 | 6.79x10^-16^ | 0.405 |
| **1.1.1.8** | 73 | 40 |  | 239 |  |  | 174.4/467.0 | <2.2x10^-16^ | 0.627 |
| **1.1.1.9** | 20 | 28 |  | 239 |  |  | 284.7/487.3 | 1.45x10^-11^ | 0.416 |
| **Unclassified** | 11 |  |  | 239 |  |  |  |  |  |
| **1.1.2** | 32 | 16 | **1.1.2** | 239 |  |  | 487.5/750.7 | 6.49x10^-12^ | 0.351 |
| **1.1.2.1** | 23 | 164 |  | 239 |  |  | 322.0/720.5 | 6.60x10^-09^ | 0.553 |
| **1.1.2.2** | 9 | 125 |  | 239 |  | 2622402 G/A | 310.0/720.5 | 4.11x10^-5^ | 0.570 |
| **1.1.3 (EAI6)** | 53 | 38 | **1.1.3** | 239 |  | - | 502.1/750.2 | <2.2x10^-16^ | 0.331 |
| **1.1.3.1** | 21 | 105 |  | 239 |  |  | 228.6/654.6 | 3.12x10^-8^ | 0.651 |
| **1.1.3.2** | 17 | 267 |  | 239 |  |  | 131.4/671.9 | 6.99x10^-7^ | 0.805 |
| **1.1.3.3** | 15 | 151 |  | 239 |  |  | 138.4/652.3 | 3.38x10^-6^ | 0.788 |
| **1.2.1 (EAI2)** | 108 | 259 | **1.2.1** | 239 |  | - | 145.3/803.0 | <2.2x10^-16^ | 0.819 |
| **1.2.1.1 (EAI2_MNL)** | 12 | 676 |  | 239 |  |  | 120.3/286.8 | 3.64x10^-05^ | 0.580 |
| **1.2.1.2 (EAI2_NTB)** | 94 | 35 |  | 239 |  |  | 106.4/272.8 | <2.2x10^-16^ | 0.610 |
| **1.2.1.3** | 2 | 67 |  | 239 |  |  | 76.0/201.9 | 0.2207* | 0.614 |
| **1.2.2** | 18 | 187 | **1.2.2** | 239 |  | 3470377 C/T | 430.2/851.9 | 3.22x10^-07^ | 0.495 |
| **2** | 521 | 99 | **2** | 105(e) |  | 497491 G/A |  |  |  |
| **2.1** | 12 | 217 | **2.1** | 105e |  | 3309880 G/A, 1881090 C/T | 265.4/727.8 | 7.4x10^-7^ | 0.635 |
| **2.2** | 509 | 92 | **2.2** | 105, 207 |  | 2505085 G/A, 4280708 G/A | 276.2/727.8 | <2.2x10^-16^ | 0.620 |
| **2.2.1** | 500 | 72 | **2.2.1** | 105, 207, 181 |  | 2825581 T/G, 797736 C/T, 4160371 C/A | 272.2/457.3 | <2.2x10^-16^ | 0.405 |
| **2.2.1/Ancestral** | 265 |  |  | 105, 207, 181 |  |  | 265.8/320.3 |  | 0.170 |
| **2.2.1.Ancestral 2** | 64 | 17 |  | 105, 207, 181 |  | 886115 T/C | 198.8/309.5 | <2.2x10^-16^ | 0.358 |
| **Bmyc6** | 9 |  |  | 105, 207, 181 | *MutT4-48* | 1892017 T/C  4137829 C/T  4393590 C/G (*mutT4*-*48*, appear in all L2.2.1 after Ancestral 2) |  |  |  |
| **2.2.1.Ancestral 3** | 146 | 6 |  | 105, 207, 181 | *MutT4-48, ogt37* | 1477522 C/A *(ogt37)* | 200.1/304.2 | <2.2x10^-16^ | 0.342 |
| **Bmyc26** | 6 |  |  | 105, 207, 181 | *MutT4-48* | 3048912 C/G (appear in all L2.2.1 after Ancestral 3) |  |  |  |
| **2.2.1.Ancestral 4** | 40 | 26 |  | 105, 207, 181 | *MutT4-48, MutT2-58* | 1286766 G/C (*mutT2-58*, appear in Ancestral 4 and all modern Beijing strains) | 121.0/325.1 | 1.43x10^-14^ | 0.629 |
| **2.2.1/Modern** | 235 | 51 |  | 105, 207, 181 | *MutT4-48, MutT2-58, ogt1*2 | 1477596 C/T *(ogt12)* | 171.2/320.3 | <2.2x10^-16^ | 0.466 |
| **2.2.1.1 (Pacific RD150)** | 22 | 36 | 2.2.1.1 | 105, 207, 181, 150 | *MutT4-48, MutT2-58, ogt1*2 | 4248115 C/T, 1844339 A/G | 85.9/193.7 | 1.43x10^-8^ | 0.556 |
| **2.2.1.Asian African 2** | 68 | 13 |  | 105, 207, 181 | *MutT4-48, MutT2-58, ogt1*2*)* | 2376135 A/G, 2532616 G/A | 152.1/204.0 | <2.2x10^-16^ | 0.255 |
| **2.2.1.2 (Asian African 2 RD142)** | 2 | 49 | 2.2.1.2 | 105, 207, 181, 142 | *MutT4-48, MutT2-58, ogt1*2 | 1692069 A/G, 1479088 C/T,  3836274 G/A |  |  |  |
| **2.2.1.Asian African 3** | 29 | 34 |  | 105, 207, 181 | *MutT4-48, MutT2-58, ogt1*2 | 1059643 G/A | 112.9/203.8 | 6.5x10^-11^ | 0.446 |
| **2.2.1. Bmyc22** | 21 | 30 |  | 105, 207, 181 | *MutT4-48, MutT2-58, ogt1*2 | 720902 G/A  (*recD277*) | 49.8/184.4 | 3.1x10^-8^ | 0.730 |
| **Bmyc20** | 1 |  |  | 105, 207, 181 | *MutT4-48, MutT2-58, ogt1*2*)* | 3048629 C/T  (*recX153*),  4027270 T/C  (*radA276*) |  |  |  |
| **2.2.1. Unclassified modern** | 92 |  |  | 105, 207, 181 | *MutT4-48, MutT2-58, ogt1*2 |  |  |  | 0.181 |
| **2.2.2 (Ancestral 1)** | 9 | 78 | **2.2.2** | 105, 207 |  | 1047873 A/C, 346693 G/T | 253.2/457.3 | 4.11x10^-5^ | 0.446 |
| **3** | 11 | 233 | **3** | 750 |  | 3273107 C/A |  |  |  |
| **4** | 523 | 126 | **4** | Pks1/15 |  | 931123 T/C |  |  |  |
| **4.1** | 8 | 79 | **4.1** | Pks1/15 |  | 62657G/A | 326.6/676.0 | 1.55x10^-4^ | 0.517 |
| **4.1.1** | 2 | 49 | **4.1.1** | Pks1/15 |  | 514245 C/T |  |  |  |
| **4.1.1.1** | 1 |  | **4.1.1.1** | Pks1/15 |  | 1850119 C/T |  |  |  |
| **4.1.1.3** | 1 |  | **4.1.1.3** | Pks1/15 |  | 4229087 C/T |  |  |  |
| **4.1.2** | **6** | 51 | **4.1.2** | Pks1/15 |  | 891756 A/G |  |  |  |
| **4.1.2.1** | 2 |  | **4.1.2.1** | Pks1/15 |  | 107794 C/T |  |  |  |
| **4.2** | 10 | 146 | **4.2** | Pks1/15 |  | 2411730 G/C | 272.6/706.8 | 1.08x10^-5^ | 0.614 |
| **4.3** | 3 | 116 | **4.3** | Pks1/15 |  | 764995 C/G | 268.7/619.8 | 0.1 | 0.567 |
| **4.3.3** | 1 |  | **4.3.3** | Pks1/15 |  | 403364 G/A |  |  |  |
| **4.3.4.2** | 2 |  | **4.3.4.2** | Pks1/15 |  | 1132368 C/T |  |  |  |
| **4.4** | 38 | 46 | **4.4** | Pks1/15 |  | 4307886 G/A | 254.3/605.5 | 6.6x10^-14^ | 0.580 |
| **4.4.1.1** | 1 |  | **4.4.1.1** | Pks1/15 |  | 355181 G/A |  |  |  |
| **4.4.2** | 37 |  | **4.4.2** | Pks1/15 |  | 4246508 G/A |  |  |  |
| **4.5** | 93 | 119 | **4.5** | Pks1/15 |  | 1719757 G/T | 313.3/628.2 | <2.2x10^-16^ | 0.501 |
| **4.5.2** | 74 | 17 |  | Pks1/15 |  |  | 286.2/391.1 | <2.2x10^-16^ | 0.268 |
| **4.5.3** | 19 | 38 |  | Pks1/15 |  |  | 272.2/391.1 | 5.66x10^-11^ | 0.304 |
| **4.8** | 6 | 244 | **4.8** | Pks1/15 |  | 3836739 G/A | 68.0/617.3 | 4.92x10^-3^ | 0.890 |

**Supplementary Table S2.** Lists of specific SNPs for each sublineage in this study. The specific SNPs displayed in green were the same as Coll’s report^5^. The specific SNP for L1 was previously reported^2^. 41 of 51 specific SNPs for Modern Beijing strains in this study are the same as the specific SNPs reported by Liu^6^.

File: Palittapongarnpim Table S2 LSSNVs.xlsx

**Supplementary Table S3** Numbers of isolates belonging to L2-L4 having various spoligotypes in Table S3A-C.

**Table S3A** Numbers of isolates belonging to sublineages of L2 having various spoligotypes. All members of few sublineages, such as Asian African 3 or Bmyc22 had exclusively the SIT1 spoligotype.

|  | SIT | Numbers of isolates in SITVITWEB^7^ | 2.1 | 2.2.2 | Asia Ancestral 2 | Asia Ancestral 3 | Ancestral 4 (new) | 2.2.1 (Bmyc6) | 2.2.1 (Bmyc26) | Asian African 2 | Asian African2/RD142 | Asian African 3 | Pacific RD150 | Bmyc22 | Unclassified Modern | Total | % of L2 |
| --- | --- | --- | --- | --- | --- | --- | --- | --- | --- | --- | --- | --- | --- | --- | --- | --- | --- |
| 000000000000371 | 250 | 24 |  |  |  |  | 3 | 1 |  |  |  |  |  |  |  | 4 | 0.77 |
| 000000000000771 | 269 | 57 |  |  |  | 21 |  |  |  |  |  |  |  |  |  | 21 | 4.03 |
| 000000000003171 | 260 | 6 |  | 1 |  | 6 |  |  |  |  |  |  |  |  |  | 7 | 1.34 |
| 000000000003371 | 265 | 42 |  |  |  | 1 |  |  |  |  |  |  |  |  |  | 1 | 0.19 |
| 000000000003611 | 3202 | 4 |  |  |  |  | 1 |  |  |  |  |  |  |  |  | 1 | 0.19 |
| 000000000003671 | 255 | 20 |  |  | 2 |  |  |  |  | 2 |  |  | 1 |  |  | 5 | 0.96 |
| 000000000003700 | 1311 | 4 |  |  | 1 |  |  |  | 1 |  |  |  |  |  |  | 2 | 0.38 |
| 000000000003731 | 190 | 76 |  |  |  | 2 | 2 |  |  |  |  |  |  |  | 4 | 8 | 1.54 |
| 000000000003761 | 1674 | 4 |  |  |  |  |  |  |  |  |  |  | 1 |  |  | 1 | 0.19 |
| 000000000003771 | 1 | Many |  | 8 | 57 | 116 | 34 | 8 | 5 | 66 | 2 | 29 | 20 | 22 | 90 | 455 | 87.33 |
| 000000007777731 | 1487 | 3 | 1 |  |  |  |  |  |  |  |  |  |  |  |  | 1 | 0.19 |
| 777777777777331 | 1149 | 6 | 1 |  |  |  |  |  |  |  |  |  |  |  |  | 1 | 0.19 |
| 777777777777771 | 523 | 27 | 10 |  | 4 |  |  |  |  |  |  |  |  |  |  | 14 | 2.69 |
| Total |  |  | 12 | 9 | 64 | 146 | 40 | 9 | 6 | 68 | 2 | 29 | 22 | 22 | 94 | 521 | 100.00 |
| % of spoligotypes other than SIT1 |  |  | 100 | 11.1 | 10.9 | 20.5 | 15.0 | 11.1 | 16.7 | 2.9 | 0 | 0 | 9.1 | 0 | 4.3 |  | 12.67 |

SIT3202 was reported in SITVIT2^8^.

**Table S3B** Spoligotypes of isolates belonging to L3.

| Spoligotypes | SIT | Clade | Number in SITVIT WEB^7^ | Number in this study |
| --- | --- | --- | --- | --- |
| 703707740003771 | 427 | CAS1-Delhi | 12 | 1 |
| 703760000000331 | 1120 | CAS | 6 | 2 |
| 703777740000771 | 357 | CAS | 55 | 1 |
| 703777740003171 | 25 | CAS1-Delhi | 254 | 2 |
| 703777740003771 | 26 | CAS1-Delhi | 896 | 5 |

**Table S3C** Numbers of isolates belonging to sublineages of L4 having various spoligotypes. All spoligotypes could be derived from SIT53, shown in bold and italic. Spoligotypes identified only in L4.5.2 was shown in bold. SIT205 was previously reported from ethnic minority in Yunnan^9^. No other studies on the genotypes of *M. tuberculosis* from ethnic minority is known to the authors.

| Spoligotypes | SIT | Clade | Number in SITVIT WEB^7^ | 4.1 | 4.2 | 4.3 | 4.4 | 4.5.2 | 4.5.3 | 4.8 | Total | |
| --- | --- | --- | --- | --- | --- | --- | --- | --- | --- | --- | --- | --- |
| 777777607760771 | 42 | LAM9 | 1952 |  |  | 2 |  |  |  |  | | 2 |
| 477777777720771 | 655 | H3 | 33 | 1 |  |  |  |  |  |  | | 1 |
| 774000370020771 | 800 | H3 | 3 |  |  |  |  |  | 3 |  | | 3 |
| **777737777720771** | **36** | **H3** | **79** |  |  |  |  | **1** |  |  | | **1** |
| 777777377720771 | 183 | H3 | 31 |  |  |  |  |  | 3 |  | | 3 |
| 777777740020771 | 946 | H3 | 14 |  |  |  |  |  | 1 |  | | 1 |
| 777777770020771 | 742 | H3 | 34 |  |  |  |  |  | 1 |  | | 1 |
| 777777777720731 | 49 | H3 | 116 |  | 1 |  |  |  |  |  | | 1 |
| 777777777720771 | 50 | H3 | 2188 | 1 |  |  |  |  | 4 | 2 | | 7 |
| 576377777760771 | 1211 | S | 2 |  |  |  | 1 |  |  |  | | 1 |
| **737777777760771** | **205** | **T1** | **30** |  |  |  |  | **4** |  |  | | **4** |
| 757777777760771 | 154 | T1 | 75 |  |  |  | 1 |  |  |  | | 1 |
| 777577777760771 | 917 | T1 | 8 |  |  |  | 1 |  |  |  | | 1 |
| 777767777760771 | 118 | T1 | 105 |  |  |  | 2 |  |  |  | | 2 |
| 777774037760771 | Orphan | T1 | 1 |  |  |  | 1 |  |  |  | | 1 |
| **777777737760771** | **86** | **T1** | **61** |  |  |  |  | **1** |  |  | | **1** |
| **777777747760771** | **1580** | **T1** | **10** |  |  |  |  | **5** |  |  | | **5** |
| **777777776360771** | **123** | **T1** | **23** |  |  |  |  | **1** |  |  | | **1** |
| 777777777660771 | 167 | T1 | 42 |  |  |  |  |  | 2 |  | | 2 |
| **777777777760371** | **240** | **T1** | **24** |  |  |  |  | **1** |  |  | | **1** |
| **777777777760571** | **520** | **T1** | **16** |  |  |  |  | **1** |  |  | | **1** |
| **777777777760760** | **628** | **T1** | **10** |  |  |  |  | **1** |  |  | | **1** |
| ***777777777760771*** | ***53*** | ***T1*** | ***3812*** |  | ***8*** |  | ***21*** | ***39*** | ***1*** | ***4*** | | ***73*** |
| 757777777760731 | 153 | T2 | 45 |  |  |  | 3 |  |  |  | | 3 |
| 777777770760731 | 942 | T2 | 4 |  |  |  | 1 |  |  |  | | 1 |
| 777777777760131 | Orphan | T2 | 1 |  |  |  | 1 |  |  |  | | 1 |
| 777777777760731 | 52 | T2 | 526 |  | 1 |  | 2 |  |  |  | | 3 |
| **777727777760771** | **1547** | **T3** | **5** |  |  |  |  | **2** |  |  | | **2** |
| **777737707760771** | **2691** | **T3** | **3** |  |  |  |  | **1** |  |  | | **1** |
| **777737777760771** | **37** | **T3** | **243** |  |  |  |  | **9** |  |  | | **9** |
| 777777757760771 | 44 | T5 | 164 |  |  |  | 1 | 4 |  |  | | 5 |
| 777776777760601 | 137 | X2 | 959 | 1 |  |  |  |  |  |  | | 1 |
| 000000007000771 | 1262 |  | 2 |  |  | 1 |  |  |  |  | | 1 |
| 777737770000000 | 56 |  | 18 |  |  |  | 1 |  |  |  | | 1 |
| 700000000720771 | new |  |  |  |  |  |  |  | 1 |  | | 1 |
| 700337777760771 | new |  |  |  |  |  | 1 |  |  |  | | 1 |
| 774776777760771 | new |  |  | 1 |  |  |  |  |  |  | | 1 |
| 777603405760431 | new |  |  | 4 |  |  |  |  |  |  | | 4 |
| **777735777760771** | **new** |  |  |  |  |  |  | **1** |  |  | | **1** |
| 777737740020731 | new |  |  |  |  |  |  |  | 1 |  | | 1 |
| 777743777420771 | new |  |  |  |  |  | 1 |  |  |  | | 1 |
| 777772700020731 | new |  |  |  |  |  |  |  | 1 |  | | 1 |
| **777777477660731** | **new** |  |  |  |  |  |  | **3** |  |  | | **3** |
| 777777777060731 | new |  |  |  |  |  |  |  | 1 |  | | 1 |
| Total |  |  |  | 8 | 10 | 3 | 38 | 74 | 19 | 6 | | 158 |

**Supplementary Table S4.** The demographic and clinical profiles of each sublineage of *M. tuberculosis* in this study. The numbers shown in the table are the number of patients in each category unless stated otherwise. RR: risk ratios, HIV: Human Immunodeficiency Virus, RMP: rifampin, INH: isoniazid, STM: streptomycin, EMB: ethambutol, MDR: resistance to both RMP and INH. Columns displaying the information for the four main lineages are shaded in light orange. Those for the Ancestral and Modern Beijing groups of L2.2.1 are shaded in light blue. Those for individual sublineages of L2.2.1 are shaded in blue. Those for other sublineages are unshaded.

|  | **L1** | **L 1.1.1** | **L 1.1.2** | **L 1.1.3** | **L 1.2.1** | **L 1.2.2** | **L2** |  | **L 2.1** | **L 2.2.2** | **L 2.2.1** | **L 2.2.1/ Ancestral** |  | **Asia  Ancestral 2** | **Asia  Ancestral 3** | **Ancestral 4** | **Bmyc6** | **Bmyc26** | **L 2.2.1/  Modern** | **L2.2.1.1** (Pacific rd150) | **Asian  African 2** | **L2.2.1.2** (Asian African 2 RD142) | **Asian  African 3** | **Bmyc22** | **Unclassified Modern** | **L3** | **L4** | **L4.1** | **L4.2** | **L4.3** | **L4.4** | **L4.5.2** | **L4.5.3** | **4.8** | **TOTAL** |
| --- | --- | --- | --- | --- | --- | --- | --- | --- | --- | --- | --- | --- | --- | --- | --- | --- | --- | --- | --- | --- | --- | --- | --- | --- | --- | --- | --- | --- | --- | --- | --- | --- | --- | --- | --- |
| **TOTAL** | **480** | **269** | **32** | **53** | **108** | **18** | **521** |  | **12** | **9** | **500** | **265** |  | **64** | **146** | **40** | **9** | **6** | **235** | **22** | **68** | **2** | **29** | **21** | **93** | **11** | **158** | **8** | **10** | **3** | **38** | **74** | **19** | **6** | **1170** |
| **Sex** |  |  |  |  |  |  |  |  |  |  |  |  |  |  |  |  |  |  |  |  |  |  |  |  |  |  |  |  |  |  |  |  |  |  |  |
| **Male** | 354 | 198 | 27 | 37 | 78 | 14 | 357 |  | 7 | 8 | 342 | 176 |  | 42 | 103 | 24 | 4 | 3 | 166 | 16 | 47 | 2 | 21 | 17 | 63 | 9 | 110 | 5 | 7 | 2 | 23 | 49 | 18 | 6 | 830 |
| **Female** | 126 | 71 | 5 | 16 | 30 | 4 | 164 |  | 5 | 1 | 158 | 89 |  | 22 | 43 | 16 | 5 | 3 | 69 | 6 | 21 | 0 | 8 | 4 | 30 | 2 | 48 | 3 | 3 | 1 | 15 | 25 | 1 | 0 | 340 |
| **Age** |  |  |  |  |  |  |  |  |  |  |  |  |  |  |  |  |  |  |  |  |  |  |  |  |  |  |  |  |  |  |  |  |  |  |  |
| **<19** | 7 | 1 | 0 | 2 | 4 | 0 | 31 |  | 0 | 1 | 30 | 19 |  | 7 | 10 | 1 | 0 | 1 | 11 | 0 | 1 | 0 | 1 | 2 | 7 | 0 | 8 | 0 | 0 | 1 | 2 | 5 | 0 | 0 | 46 |
| **20-29** | 43 | 24 | 2 | 4 | 12 | 1 | 95 |  | 1 | 2 | 92 | 54 |  | 15 | 29 | 6 | 4 | 0 | 38 | 4 | 12 | 0 | 5 | 7 | 10 | 3 | 26 | 1 | 1 | 1 | 7 | 14 | 2 | 0 | 167 |
| **30-39** | 92 | 42 | 6 | 15 | 25 | 4 | 119 |  | 0 | 2 | 117 | 61 |  | 13 | 31 | 14 | 1 | 2 | 55 | 5 | 17 | 0 | 6 | 6 | 21 | 3 | 36 | 1 | 3 | 0 | 8 | 19 | 3 | 2 | 251 |
| **40-49** | 92 | 47 | 8 | 12 | 20 | 5 | 114 |  | 4 | 1 | 109 | 51 |  | 9 | 32 | 6 | 1 | 3 | 57 | 7 | 19 | 0 | 8 | 4 | 19 | 3 | 41 | 3 | 4 | 1 | 10 | 13 | 7 | 3 | 250 |
| **50-59** | 88 | 57 | 5 | 7 | 18 | 1 | 80 |  | 4 | 2 | 74 | 41 |  | 12 | 18 | 10 | 1 | 0 | 33 | 3 | 8 | 0 | 4 | 2 | 16 | 0 | 25 | 1 | 0 | 0 | 5 | 15 | 3 | 1 | 192 |
| **60-69** | 83 | 50 | 9 | 10 | 11 | 3 | 45 |  | 3 | 1 | 41 | 23 |  | 5 | 15 | 2 | 1 | 0 | 18 | 1 | 6 | 0 | 3 | 0 | 8 | 1 | 13 | 1 | 2 | 0 | 3 | 5 | 2 | 0 | 142 |
| **>70** | 75 | 48 | 2 | 3 | 18 | 4 | 39 |  | 0 | 0 | 39 | 16 |  | 3 | 11 | 1 | 1 | 0 | 23 | 2 | 5 | 2 | 2 | 0 | 12 | 1 | 9 | 1 | 0 | 0 | 3 | 3 | 2 | 0 | 124 |
| **Average Ages**  **(years)** | 51.1 | 53.4 | 50.3 | 45.6 | 48.5 | 51.9 | 42.3 |  | 51.6 | 39.7 | 42.2 | 40.9 |  | 39.2 | 41.9 | 40.6 | 41.8 | 35.3 | 43.6 | 44 | 43.1 | 73.5 | 44.2 | 33.9 | 43.3 | 41.4 | 42.2 | 47.5 | 43.9 | 28 | 42.8 | 40.6 | 46.7 | 42.5 | 45.9 |
| **SD** | (16.7) | (16.4) | (14) | (15.8) | (17.7) | (16.9) | (15.9) |  | (10.8) | (15.5) | (15.9) | (15.7) |  | (16) | (16.6) | (12.2) | (17.6) | (9.9) | (16.1) | (16) | (14.9) | (4.9) | (15.6) | (10.8) | (16.5) | (15.5) | (15.2) | (14.9) | (12.9) | (12.1) | (16) | (15.3) | (16.4) | (7.7) | (16.7) |
| **Median Ages**  **(years)** | 50 | 54 | 49 | 43 | 46 | 47 | 41 |  | 52.5 | 38 | 41 | 39 |  | 37.5 | 40 | 39 | 37 | 37.5 | 42 | 42 | 41.5 | 74 | 46 | 31 | 43 | 34 | 41 | 41 | 42.5 | 26 | 45 | 39 | 44 | 43.5 | 45 |
| **(IQR)** | (38-65) | (40-66) | (39.5-65) | (33-58) | (35-62.5) | (39-65) | (30-52) |  | (45-59.5) | (29-52) | (30-52) | (29-51) |  | (27-52) | (29-52) | (32-50) |  |  | (30-54) | (33-52) | (31-53) | (70-77) | 30-51 | 28-43 | (30-53) | (29-49) | (32-52) | (36.5-59) | (34-48) | (17-410 | (33-54) | (29-52) | (32-52) | (36-47) | (33-58) |
| **% with ages >49** | 51.3 | 57.6 | 50.0 | 37.7 | 43.5 | 44.4 | 31.5 |  | 58.3 | 33.3 | 30.8 | 30.2 |  | 31.3 | 30.1 | 32.5 | 33.3 | 0.0 | 31.5 | 27.3 | 27.9 | 100.0 | 31.0 | 9.5 | 50.5 | 18.2 | 29.1 | 37.5 | 20.0 | 0.0 | 28.9 | 31.1 | 36.8 | 16.7 | 39.1 |
| **Ethnicity** |  |  |  |  |  |  |  |  |  |  |  |  |  |  |  |  |  |  |  |  |  |  |  |  |  |  |  |  |  |  |  |  |  |  |  |
| **Thai** | 401 | 223 | 23 | 40 | 99 | 16 | 282 |  | 9 | 5 | 268 | 113 |  | 25 | 70 | 12 | 1 | 5 | 155 | 21 | 44 | 2 | 20 | 11 | 57 | 4 | 66 | 4 | 7 | 2 | 22 | 18 | 10 | 3 | 753 |
| **Other Tai ethnic groups** | 15 | 7 | 3 | 2 | 3 | 0 | 30 |  | 0 | 0 | 30 | 21 |  | 6 | 10 | 1 | 4 | 0 | 9 | 0 | 4 | 0 | 0 | 1 | 4 | 1 | 11 | 1 | 1 | 0 | 1 | 5 | 3 | 0 | 57 |
| **RR** | 0.49 (0.32-0.77) | 0.41 (0.21-0.84) | 1.72 | 0.66 | 0.40 | 0 | 1.41 (1.08-1.83) |  | 0 | 0 | 1.48 (1.14-1.93) | 2.46 (1.68-3.59) |  | 3.17 (1.36-7.41) | 1.89 (1.03-3.46) | 1.10 | 52.84 (6.0-465.0) | 0 | 0.77 | 0 | 1.20 | 0 | 0 | 1.20 | 0.93 | 3.30 | 2.20 (1.23-3.93) | 3.30 | 1.89 | 0 | 0.60 | 3.67 (1.41-9.52) | 3.96 (1.12-14.0) | 0 |  |
| **Chinese** | 0 | 0 | 0 | 0 | 0 | 0 | 9 |  | 0 | 0 | 9 | 5 |  | 1 | 3 | 0 | 1 | 0 | 4 | 0 | 2 | 0 | 0 | 1 | 1 | 0 | 3 | 0 | 0 | 0 | 1 | 1 | 1 | 0 | 12 |
| **RR** | 0 | 0 | 0 | 0 | 0 | 0 | 2.00 (1.43-2.81) |  | 0 | 0 | 2.11 (1.50-2.96) | 2.78 (1.39-5.54) |  | 2.51 | 2.69 | 0.00 | 62.75 (4.16-945.5) | 0 | 1.62 | 0 | 2.85 | 0 | 0 | 5.70 | 1.10 | 0 | 2.85 (1.04-7.81) | 0 | 0 | 0 | 2.85 | 3.49 | 6.28 | 0 |  |
| **Burman** | 5 | 0 | 1 | 3 | 1 | 0 | 21 |  | 0 | 1 | 20 | 14 |  | 5 | 7 | 0 | 2 | 0 | 6 | 0 | 2 | 0 | 0 | 1 | 3 | 2 | 2 | 0 | 0 | 0 | 0 | 1 | 1 | 0 | 30 |
| **RR** | 0.31 (0.14-0.70) | 0 | 1.09 | 1.88 | 0.25 | 0 | 1.87 (1.45-2.40) |  | 0 | 5.02 | 1.87 (1.43-2.46) | 3.11 (2.05-4.73) |  | 5.02 (2.07-17.05) | 2.51 (1.26-4.98) | 0.00 | 50.20 (4.68-538.4) | 0 | 0.97 | 0 | 1.14 | 0 | 0 | 2.28 | 1.32 | 12.55 (2.39-65.85) | 0.76 | 0 | 0 | 0 | 0 | 1.39 | 2.51 | 0 |  |
| **Akha** | 23 | 15 | 3 | 3 | 2 | 0 | 83 |  | 1 | 1 | 81 | 51 |  | 9 | 26 | 15 | 1 | 0 | 30 | 0 | 7 | 0 | 4 | 4 | 15 | 1 | 41 | 1 | 0 | 1 | 9 | 29 | 0 | 0 | 148 |
| **RR** | 0.29 (0.20-0.43) | 0.35 (0.21-0.57) | 0.67 | 0.38 | 0.10 (0.03-0.41) | 0 | 1.50 (1.26-1.77) |  | 0.57 | 1.02 | 1.54 (1.29-1.83) | 2.30 (1.74-3.04) |  | 1.84 | 1.89 (1.25-2.86) | 6.36 (3.04-13.31) | 5.12 | 0 | 0.99 | 0 | 0.81 | 0 | 1.02 | 1.86 | 1.35 | 1.28 | 3.16 (2.23-4.47) | 1.28 | 0 | 2.56 | 2.10 | 8.20 (4.68-14.37) | 0 | 0 |  |
| **Lahu** | 7 | 5 | 0 | 1 | 1 | 0 | 47 |  | 0 | 1 | 46 | 29 |  | 10 | 12 | 6 | 0 | 1 | 17 | 0 | 5 | 0 | 1 | 1 | 10 | 1 | 20 | 0 | 1 | 0 | 3 | 12 | 3 | 1 | 75 |
| **RR** | 0.18 (0.09-0.36) | 0.23 (0.10-0.53) | 0 | 0.25 | 0.10 (0.01-0.72) | 0 | 1.67 (1.37-2.04) |  | 0 | 2.01 | 1.72 (1.41-2.11) | 2.58 (1.85-3.59) |  | 4.02 (2.01-8.04) | 1.72 | 5.02 (1.94-12.99) | 0 | 2.01 | 1.10 | 0 | 1.14 | 0 | 0.50 | 0.91 | 1.76 | 2.51 | 3.04 (1.96-4.73) | 0.00 | 1.43 | 0.00 | 1.37 | 6.69 (3.35-13.36) | 3.01 | 3.35 |  |
| **Other Tibeto-Burman family** | 2 | 0 | 0 | 1 | 0 | 1 | 5 |  | 0 | 0 | 5 | 4 |  | 1 | 2 | 1 | 0 | 0 | 1 | 0 | 0 | 0 | 0 | 0 | 1 | 0 | 0 | 0 | 0 | 0 | 0 | 0 | 0 | 0 | 7 |
| **RR** | 0.54 | 0 | 0 | 2.69 | 0.00 | 6.72 (1.03-43.98) | 1.91 (1.18-3.07) |  | 0 | 0 | 2.01 (1.24-3.24) | 3.81 (1.96-7.39) |  | 4.30 | 3.07 | 8.96 (1.34-59.90) | 0 | 0 | 0.69 | 0 | 0 | 0 | 0 | 0 | 1.89 | 0.00 | 0.00 | 0 | 0 | 0 | 0 | 0 | 0 | 0 |  |
| **All Tibeto-Burman** | 37 | 20 | 4 | 8 | 4 | 1 | 156 |  | 1 | 3 | 152 | 98 |  | 25 | 47 | 22 | 3 | 1 | 54 | 0 | 14 | 0 | 5 | 6 | 29 | 4 | 62 | 1 | 1 | 1 | 12 | 42 | 4 | 1 | 260 |
| **RR** | 0.27 (0.20-0.37) | 0.29 (0.17-0.41) | 0.43 | 0.58 | 0.12 (0.03-0.31) | 0.18 | 1.60 (1.40-1.83) |  | 0.32 | 1.74 | 1.64 (1.43-1.89) | 2.51 (1.99-3.16) |  | 2.90 (1.69-4.95) | 1.94 (1.38-2.74) | 5.31 (2.67-10.58) | 8.69 | 0.58 | 1.01 | 0 | 0.92 | 0 | 0.72 | 1.58 | 1.47 | 2.90 | 2.76 (2.02-3.79) | 0.72 | 0.41 | 1.45 | 1.71 | 6.76 (3.96-11.53) | 1.16 | 0.97 |  |
| **Hmong-Mien** | 3 | 1 | 1 | 1 | 0 | 0 | 9 |  | 0 | 1 | 8 | 7 |  | 1 | 4 | 2 | 0 | 0 | 1 | 0 | 0 | 0 | 1 | 0 | 0 | 1 | 3 | 0 | 0 | 0 | 0 | 2 | 1 | 0 | 16 |
| **RR** | 0.35 (0.13-0.98) | 0.21 | 2.05 | 1.18 | 0 | 0 | 1.50 |  | 0.00 | 9.41 (1.17-76.04) | 1.40 | 2.92 (1.63-5.11) |  | 1.88 | 2.69 (1.12-6.47) | 7.84 (1.91-32.21) | 0 | 0 | 0.30 | 0 | 0 | 0 | 2.35 | 0 | 0 | 11.77 (1.39-99.47) | 2.14 | 0 | 0 | 0 | 0 | 5.23 (1.32-20.67) | 4.71 | 0 |  |
| **Other ethnic minority groups** | 5 | 3 | 1 | 1 | 0 | 0 | 11 |  | 0 | 0 | 11 | 8 |  | 4 | 3 | 1 | 0 | 0 | 3 | 0 | 2 | 0 | 0 | 0 | 1 | 0 | 5 | 1 | 1 | 0 | 1 | 2 | 0 | 0 | 21 |
| **RR** | 0.45 (0.21-0.96) | 0.48 | 1.56 | 0.90 | 0 | 0 | 1.40 |  | 0 | 0 | 1.47 | 2.54 (1.43-4.49) |  | 5.74 (2.19-15.02) | 1.54 | 2.99 | 0 | 0 | 0.69 | 0 | 1.63 | 0 | 0 | 0 | 0.63 | 0 | 2.71 (1.22-6.04) | 8.96 | 5.12 | 0.00 | 1.63 | 3.98 | 0 | 0 |  |
| **Non- Thai** | 60 | 31 | 9 | 12 | 7 | 1 | 215 |  | 1 | 4 | 210 | 139 |  | 37 | 67 | 26 | 8 | 1 | 71 | 0 | 22 | 0 | 6 | 8 | 35 | 6 | 84 | 3 | 3 | 1 | 15 | 52 | 9 | 1 | 365 |
| **RR** | 0.31 (0.24-0.39) | 0.29 (0.20-0.41) | 0.81 | 0.62 | 0.15 (0.07-0.31) | 0.13 (0.02-0.97) | 1.57 (1.39-1.78) |  | 0.23 | 1.65 | 1.62 (1.42-1.84) | 2.54 (2.05-3.14) |  | 3.05 (1.87-4.99) | 1.97 (1.45-2.69) | 4.47 (2.28-8.76) | 16.50 | 0.41 | 0.94 | 0 | 1.03 | 0 | 0.62 | 1.50 | 1.27 | 3.09 | 2.63 (1.95-3.53) | 1.55 | 0.88 | 1.03 | 1.41 | 5.96 (3.54-10.04) | 1.86 | 0.69 |  |
| **Clinical presentations** |  |  |  |  |  |  |  |  |  |  |  |  |  |  |  |  |  |  |  |  |  |  |  |  |  |  |  |  |  |  |  |  |  |  |  |
| **Pulmonary** | 458 | 258 | 32 | 49 | 102 | 17 | 497 |  | 11 | 9 | 477 | 255 |  | 62 | 140 | 39 | 8 | 6 | 222 | 20 | 64 | 2 | 26 | 20 | 90 | 11 | 156 | 8 | 10 | 3 | 36 | 74 | 19 | 6 | 1122 |
| **Pulmonary and Extra-pulmonary** | 20 | 9 | 0 | 4 | 6 | 1 | 23 |  | 1 | 0 | 22 | 10 |  | 2 | 6 | 1 | 1 | 0 | 12 | 2 | 4 | 0 | 3 | 0 | 3 | 0 | 2 | 0 | 0 | 0 | 2 | 0 | 0 | 0 | 45 |
| **Extra-pulmonary** | 2 | 2 | 0 | 0 | 0 | 0 | 1 |  | 0 | 0 | 1 | 0 |  | 0 | 0 | 0 | 0 | 0 | 1 | 0 | 0 | 0 | 0 | 1 | 0 | 0 | 0 | 0 | 0 | 0 | 0 | 0 | 0 | 0 | 3 |
| **HIV status** |  |  |  |  |  |  |  |  |  |  |  |  |  |  |  |  |  |  |  |  |  |  |  |  |  |  |  |  |  |  |  |  |  |  |  |
| **Positive** | 99 | 49 | 5 | 12 | 29 | 4 | 77 |  | 1 | 2 | 74 | 38 |  | 9 | 23 | 3 | 1 | 2 | 36 | 3 | 11 | 0 | 4 | 8 | 10 | 1 | 22 | 3 | 2 | 0 | 6 | 5 | 4 | 2 | 199 |
| **Negative** | 371 | 215 | 27 | 40 | 76 | 13 | 439 |  | 11 | 7 | 421 | 225 |  | 54 | 122 | 37 | 8 | 4 | 196 | 18 | 55 | 2 | 25 | 13 | 83 | 9 | 135 | 5 | 8 | 3 | 31 | 69 | 15 | 4 | 954 |
| **Unknown** | 10 | 5 | 0 | 1 | 3 | 1 | 5 |  | 0 | 0 | 5 | 2 |  | 1 | 1 | 0 | 0 | 0 | 3 | 1 | 2 | 0 | 0 | 0 | 0 | 1 | 1 | 0 | 0 | 0 | 1 | 0 | 0 | 0 | 17 |
| **% with HIV infection** | 20.625 | 18.6 | 15.6 | 23.1 | 27.6 | 23.5 | 14.9 |  | 8.3 | 22.2 | 14.9 | 14.4 |  | 14.3 | 15.9 | 7.5 | 11.1 | 33.3 | 15.5 | 14.3 | 16.7 | 0 | 13.8 | 38.1 | 10.8 | 10 | 14.0 | 37.5 | 20.0 | 0.0 | 16.2 | 6.8 | 21.1 | 33.3 | 17.3 |
| **Drug Resistance** |  |  |  |  |  |  |  |  |  |  |  |  |  |  |  |  |  |  |  |  |  |  |  |  |  |  |  |  |  |  |  |  |  |  |  |
| **Rifampin** |  |  |  |  |  |  |  |  |  |  |  |  |  |  |  |  |  |  |  |  |  |  |  |  |  |  |  |  |  |  |  |  |  |  |  |
| **Resistance** | 12 | 10 | 1 | 0 | 1 | 0 | 31 |  | 4 | 0 | 27 | 11 |  | 1 | 6 | 0 | 2 | 2 | 16 | 1 | 8 | 0 | 6 | 0 | 1 | 2 | 7 | 0 | 0 | 0 | 2 | 5 | 0 | 0 | 52 |
| **Sensitive** | 429 | 241 | 27 | 49 | 96 | 16 | 458 |  | 7 | 9 | 442 | 231 |  | 60 | 124 | 36 | 7 | 4 | 211 | 18 | 58 | 2 | 22 | 21 | 90 | 8 | 146 | 8 | 8 | 3 | 35 | 67 | 19 | 6 | 1041 |
| **% resistance** | 2.7 | 4.0 | 3.6 | 0 | 1.0 | 0 | 6.3 |  | 36.4 | 0 | 5.8 | 4.5 |  | 1.6 | 4.6 | 0.0 | 22.2 | 33.3 | 7.0 | 5.3 | 12.1 | 0.0 | 21.4 | 0.0 | 1.1 | 20.0 | 4.6 | 0.0 | 0.0 | 0.0 | 5.4 | 6.9 | 0.0 | 0.0 | 4.8 |
| **Isoniazid** |  |  |  |  |  |  |  |  |  |  |  |  |  |  |  |  |  |  |  |  |  |  |  |  |  |  |  |  |  |  |  |  |  |  |  |
| **Resistance** | 42 | 25 | 5 | 2 | 7 | 3 | 79 |  | 4 | 2 | 73 | 20 |  | 5 | 9 | 2 | 2 | 2 | 53 | 3 | 16 | 0 | 11 | 1 | 22 | 5 | 21 | 0 | 2 | 0 | 6 | 6 | 3 | 4 | 147 |
| **Sensitive** | 399 | 226 | 23 | 47 | 90 | 13 | 409 |  | 7 | 7 | 395 | 221 |  | 56 | 121 | 33 | 7 | 4 | 174 | 16 | 50 | 2 | 17 | 20 | 69 | 5 | 132 | 8 | 6 | 3 | 31 | 66 | 16 | 2 | 945 |
| **% resistance** | 9.5 | 10.0 | 17.9 | 4.1 | 7.2 | 18.8 | 16.2 |  | 36.4 | 22.2 | 15.6 | 8.3 |  | 8.2 | 6.9 | 5.7 | 22.2 | 33.3 | 23.3 | 15.8 | 24.2 | 0 | 39.3 | 4.8 | 24.2 | 50.0 | 13.7 | 0 | 25.0 | 0 | 16.2 | 8.3 | 15.8 | 66.7 | 13.5 |
| **Streptomycin** |  |  |  |  |  |  |  |  |  |  |  |  |  |  |  |  |  |  |  |  |  |  |  |  |  |  |  |  |  |  |  |  |  |  |  |
| **Resistance** | 15 | 5 | 1 | 4 | 3 | 2 | 74 |  | 2 | 1 | 71 | 19 |  | 7 | 8 | 3 | 0 | 1 | 52 | 3 | 14 | 1 | 16 | 0 | 18 | 2 | 11 | 0 | 0 | 0 | 8 | 2 | 1 | 0 | 102 |
| **Sensitive** | 426 | 246 | 27 | 45 | 94 | 14 | 415 |  | 9 | 8 | 398 | 223 |  | 54 | 122 | 33 | 9 | 5 | 175 | 16 | 52 | 1 | 12 | 21 | 73 | 8 | 142 | 8 | 8 | 3 | 29 | 70 | 18 | 6 | 991 |
| **% resistance** | 3.4 | 2.0 | 3.6 | 8.2 | 3.1 | 12.5 | 15.1 |  | 18.2 | 11.1 | 15.1 | 7.9 |  | 11.5 | 6.2 | 8.3 | 0 | 16.7 | 22.9 | 15.8 | 21.2 | 50.0 | 57.1 | 0 | 19.8 | 20.0 | 7.2 | 0 | 0 | 0 | 21.6 | 2.8 | 5.3 | 0.0 | 9.3 |
| **Ethambutol** |  |  |  |  |  |  |  |  |  |  |  |  |  |  |  |  |  |  |  |  |  |  |  |  |  |  |  |  |  |  |  |  |  |  |  |
| **Resistance** | 2 | 1 | 0 | 0 | 1 | 0 | 12 |  | 1 | 0 | 11 | 3 |  | 1 | 1 | 0 | 0 | 1 | 8 | 2 | 3 | 0 | 3 | 0 | 0 | 1 | 3 | 0 | 0 | 0 | 1 | 2 | 0 | 0 | 18 |
| **Sensitive** | 439 | 250 | 28 | 49 | 96 | 16 | 477 |  | 10 | 9 | 458 | 239 |  | 60 | 129 | 36 | 9 | 5 | 219 | 17 | 63 | 2 | 25 | 21 | 91 | 9 | 150 | 8 | 8 | 3 | 36 | 70 | 19 | 6 | 1075 |
| **% resistance** | 0.5 | 0.4 | 0 | 0 | 1.0 | 0 | 2.5 |  | 9.1 | 0 | 2.3 | 1.2 |  | 1.6 | 0.8 | 0 | 0 | 16.7 | 3.5 | 10.5 | 4.5 | 0 | 10.7 | 0 | 0 | 10.0 | 2.0 | 0 | 0 | 0 | 2.7 | 2.8 | 0 | 0 | 1.6 |
| **MDR** | 10 | 9 | 0 | 0 | 1 | 0 | 23 |  | 3 | 0 | 20 | 5 |  | 0 | 2 | 0 | 2 | 1 | 15 | 1 | 8 | 0 | 5 | 0 | 1 | 2 | 6 | 0 | 0 | 0 | 2 | 4 | 0 | 0 | 41 |
| **% MDR** | **2.27** | **3.59** | **0** | **0** | **1.03** | **0** | **4.70** |  | **27.3** | **0.00** | **4.26** | **2.07** |  | **0.00** | **1.54** | **0** | **22.2** | **16.7** | **6.61** | **5.26** | **12.1** | **0** | **17.9** | **0** | **1.10** | **20.0** | **3.92** | **0** | **0** | **0** | **5.41** | **5.56** | **0** | **0** | **3.75** |

**Supplementary Table S5** Nonsynonymous SNVs specific to sublineage L2.2.1.Asian African 3. The functional category was classified according to Mycobrowser database (https://mycobrowser.epfl.ch/). Essentiality refers to the necessity for *M. tuberculosis* survival; E = essential gene, NE = non-essential gene. Mutational effects on protein function were predicted by two algorithms (Polyphen-1 and SIFT); N = neutral (no effect), A = affected.

| **Functional category** | **Gene number** | **Gene name** | **Amino acid change** | **Essentiality** | **Polyphen-1** | **STFT** | **Functional annotation** |
| --- | --- | --- | --- | --- | --- | --- | --- |
| **Cell wall and cell processes** | **Rv0236c** | **aftD** | **Gly755Glu** | **E** | **A** | **A** | **Arabinofuranosyltransferase AftD, involved in the biosynthesis of the mycobacterial cell wall arabinan** |
|  | Rv0658c | Rv0658c | Gly42Asp | NE | A | N | Possible integral membrane protein |
|  | Rv3479 | Rv3479 | Phe446Leu | NE | N | N | Possible transmembrane protein |
|  | **Rv3689** | **Rv3689** | **Tyr410Ser** | **NE** | **A** | **A** | **Probable transmembrane protein** |
| **Conserved hypotheticals** | Rv0140 | Rv0140 | Val6Met | NE | N | A | Hypothetical protein |
|  | Rv1290c | Rv1290c | Ala481Ser | NE | A | N | Hypothetical protein |
|  | Rv2022c | Rv2022c | Asp20Asn | NE | N | N | Hypothetical protein |
|  | **Rv2230c** | **Rv2230c** | **Arg260Cys** | **NE** | **A** | **A** | **Hypothetical protein** |
|  | Rv2897c | Rv2897c | Asp15Gly | E | N | N | Hypothetical protein, possibly Mg2+ chelatase |
| **Information pathways** | Rv0949 | uvrD1 | Gly462Ser | E | N | N | Probable ATP-dependent DNA helicase II |
|  | Rv3241c | Rv3241c | Gly136Arg | NE | N | N | Hypothetical protein |
| **Intermediary metabolism and respiration** | Rv0687 | Rv0687 | Val74Leu | NE | N | A | Probable dehydrogenase/reductase, involved in cellular metabolism |
|  | **Rv1122** | **gnd2** | **Asp318Tyr** | **E** | **A** | **A** | **6-phosphogluconate dehydrogenase (enzyme in pentose phosphate pathway)** |
|  | Rv3227 | aroA | Ala88Thr | E | N | N | 3-phosphoshikimate 1-carboxyvinyltransferase, biosynthesis of aromatic amino acids |
| **Regulatory proteins** | Rv0737 | Rv0737 | Asp164Glu | NE | N | A | Possible transcriptional regulatory protein |
| **Virulence, detoxification, adaptation** | Rv0169 | mce1A | Lys63Glu | NE | N | A | Mce1-family protein, related to cell entry and invasion of into mammalian cells |
|  | **Rv3063** | **cstA** | **Arg314His** | **NE** | **A** | **A** | **Probable carbon starvation protein A homolog** |
| **Lipid metabolism** | **Rv2950c** | **fadD29** | **Arg600Gly** | **NE** | **A** | **A** | **Fatty-acid-AMP ligase FadD29** |
|  | **Rv1425** | **Rv1425** | **Gly128Cys** | **NE** | **A** | **A** | **Possible triacylglycerol synthase** |

**REFERENCES**

1 Zhang, H. *et al.* Genome sequencing of 161 Mycobacterium tuberculosis isolates from China identifies genes and intergenic regions associated with drug resistance. *Nat Genet* **45**, 1255-1260, doi:10.1038/ng.2735 (2013).

2 Palittapongarnpim, P. *et al.* Evidence for Host-Bacterial Co-evolution via Genome Sequence Analysis of 480 Thai Mycobacterium tuberculosis Lineage 1 Isolates. *Sci Rep* **8**, 11597, doi:10.1038/s41598-018-29986-3 (2018).

3 Shitikov, E. *et al.* Evolutionary pathway analysis and unified classification of East Asian lineage of Mycobacterium tuberculosis. *Sci Rep* **7**, 9227, doi:10.1038/s41598-017-10018-5 (2017).

4 Mestre, O. *et al.* Phylogeny of Mycobacterium tuberculosis Beijing strains constructed from polymorphisms in genes involved in DNA replication, recombination and repair. *PLoS One* **6**, e16020, doi:10.1371/journal.pone.0016020 (2011).

5 Coll, F. *et al.* A robust SNP barcode for typing Mycobacterium tuberculosis complex strains. *Nat Commun* **5**, 4812, doi:10.1038/ncomms5812 (2014).

6 Liu, Q. *et al.* Genetic features of Mycobacterium tuberculosis modern Beijing sublineage. *Emerg Microbes Infect* **5**, e14, doi:10.1038/emi.2016.14 (2016).

7 Demay, C. *et al.* SITVITWEB--a publicly available international multimarker database for studying Mycobacterium tuberculosis genetic diversity and molecular epidemiology. *Infect Genet Evol* **12**, 755-766, doi:10.1016/j.meegid.2012.02.004 (2012).

8 Couvin, D. & Rastogi, N. Tuberculosis - A global emergency: Tools and methods to monitor, understand, and control the epidemic with specific example of the Beijing lineage. *Tuberculosis (Edinb)* **95 Suppl 1**, S177-189, doi:10.1016/j.tube.2015.02.023 (2015).

9 Chen, L. *et al.* First Insight into the Molecular Epidemiology of Mycobacterium tuberculosis Isolates from the Minority Enclaves of Southwestern China. *Biomed Res Int* **2017**, 2505172, doi:10.1155/2017/2505172 (2017).
